# Immunological analysis of LC16m8 vaccine: preclinical and early clinical insights into mpox

**DOI:** 10.1016/j.ebiom.2025.105703

**Published:** 2025-04-15

**Authors:** Kouji Kobiyama, Daichi Utsumi, Yu Kaku, Eita Sasaki, Fumihiko Yasui, Tomotaka Okamura, Taishi Onodera, Asuka Joy Tobuse, Areej Sakkour, Ahmad Faisal Amiry, Tomoya Hayashi, Burcu Temizoz, Kaiwen Liu, Hideo Negishi, Noriko Toyama-Sorimachi, Michinori Kohara, Tatsuya Sawasaki, Junichi Takagi, Kei Sato, Yoshimasa Takahashi, Yasuhiro Yasutomi, Ken J. Ishii

**Affiliations:** a Division of Vaccine Science, The Institute of Medical Science, The University of Tokyo, Tokyo, Japan; b International Vaccine Design Center, The Institute of Medical Science, The University of Tokyo, Tokyo, Japan; c Laboratory of Immunoregulation and Vaccine Research, Tsukuba Primate Research Center, National Institutes of Biomedical Innovation, Health and Nutrition, Ibaraki, Japan; d Division of Systems Virology, The Institute of Medical Science, The University of Tokyo, Tokyo, Japan; e Research Center for Drug and Vaccine Development, National Institute of Infectious Diseases, Tokyo, Japan; f Department of Microbiology and Cell Biology, Tokyo Metropolitan Institute of Medical Science, Tokyo, Japan; g Division of Human Immunology, International Vaccine Design Center, The Institute of Medical Science, The University of Tokyo, Tokyo, Japan; h Proteo-Science Center (PROS), Ehime University, Matsuyama, Japan; i Laboratory for Protein Synthesis and Expression, Institute for Protein Research, Osaka University, Osaka, Japan; j International Research Center for Infectious Diseases, The Institute of Medical Science, The University of Tokyo, Tokyo, Japan; k Graduate School of Medicine, The University of Tokyo, Tokyo, Japan; l Department of Computational Biology and Medical Sciences, Graduate School of Frontier Sciences, The University of Tokyo, Kashiwa, Japan; m Collaboration Unit for Infection, Joint Research Center for Human Retrovirus Infection, Kumamoto University, Kumamoto, Japan; n MRC-University of Glasgow Centre for Virus Research, Glasgow, UK; o The University of Tokyo Pandemic Preparedness, Infection and Advanced Research Center (UTOPIA), The University of Tokyo, Tokyo, Japan

**Keywords:** Mpox, LC16m8 vaccine, Immunological evaluation, Antigen-specific immunity

## Abstract

**Background:**

The global mpox outbreak (2022–2024) highlights the need for effective and safe vaccines, particularly for vulnerable populations. The LC16m8 vaccine, an attenuated vaccinia virus strain for smallpox, shows promise in inducing immunity against the monkeypox virus (MPXV).

**Methods:**

We conducted a comprehensive immunological evaluation of LC16m8 in mice, non-human primates, and humans.

**Findings:**

LC16m8 induced strong humoural responses in BALB/c, C57BL/6J, and CAST/EiJ mice, targeting MPXV H3, A35, and M1R antigens, promoting germinal centre B cells and follicular helper T cells, essential for long-term immunity. Vaccinated CAST/EiJ mice showed reduced lung MPXV viral loads, demonstrating efficacy. In humans, LC16m8 enhanced neutralising antibodies against multiple MPXV clades, suggesting broad protection. In cynomolgus monkeys, systemic administration caused localised pox lesions without significantly affecting weight, temperature, or haematological parameters.

**Interpretation:**

This cross-species immunological analysis provides preclinical and early clinical insights into LC16m8's efficacy and safety against mpox. While LC16m8 enhanced antibody responses against MPXV clade Ia and Ib, further studies are required to evaluate its efficacy, particularly in naive and immunocompromised populations.

**Funding:**

This research was supported by 10.13039/100009619AMED under Grant Numbers JP243fa727002, JP243fa727001s0703, and JP243fa627001h0003 (K.J.I), JP24jf0126002, JP24fk0108690, JP243fa627001h0003, and JP243fa727002 (K.S), JP243fa727002 (Y.T.), JP243fa727002 and JP243fa627007h0003 (Y.Y.), and by the Research Support Project for Life Science and Drug Discovery (BINDS) from 10.13039/100009619AMED under Grant Number JP23ama121011 (J.T.), and JP23ama121010 (T.S.), and by the 10.13039/501100001700Ministry of Education, Culture, Sports, Science and Technology in Japan under Grant Number 23K06577 (E.S.). 10.13039/100009619AMED under Grant Number JP233fa827017 and JP243fa827017 (F.Y.), JP22fk0108501 (M.K.).

**Research in context**

**Evidence before this study**

LC16m8, an attenuated vaccinia virus strain, was initially developed in Japan for smallpox and approved for monkeypox (MPXV) in 2022. Previous studies demonstrated the efficacy of LC16m8 against MPXV in non-human primates, establishing it as a key medical countermeasure. However, these studies primarily focused on viral load reduction and survival, providing limited insight into specific antigenic targets, cellular immune responses, and immunogenicity across MPXV clades, particularly the outbreak-associated clade Ib. In addition, LC16m8 efficacy in a mouse model remains undetermined.

**Added value of this study**

This study systematically evaluated the immunogenicity and efficacy of LC16m8 in preclinical mouse models and recently vaccinated individuals. Using three genetically distinct mouse strains (BALB/c, C57BL/6J, and CAST/EiJ), we examined variations in antibody and T-cell responses. LC16m8 induced selective antibodies against key MPXV antigens (e.g. H3, A35, and M1) and revealed differential antigen immunogenicity, providing insights for serological diagnostics and vaccine optimisation. We demonstrated the robust induction of germinal centre B cells and follicular helper T cells, highlighting the vaccine's ability to elicit strong humoural and cellular immunity. In humans, LC16m8 enhanced antibody responses against multiple MPXV clades, demonstrating comparable immunogenicity across Clades Ia and Ib despite a single mutation in the A35 protein. These findings underscore the vaccine's broad protective potential and relevance in addressing MPXV genetic diversity.

**Implications of all the available evidence**

LC16m8 is a promising vaccine candidate against MPXV, particularly amid the ongoing Public Health Emergency of International Concern (PHEIC). Its ability to induce durable humoural and cellular immunity, coupled with a favourable safety profile, makes it effective for global immunisation. Future studies should focus on optimising formulations to enhance immunogenicity across diverse demographic and genetic populations. Moreover, incorporating complementary approaches, such as mRNA or protein subunit vaccines, may improve vaccine safety and scalability. Ensuring vaccine accessibility through international collaboration, particularly in endemic regions, is critical for effective outbreak control and prevention.

## Introduction

Emerging infectious diseases continue to pose a major global health threat, as demonstrated by outbreaks of H1N1 influenza virus (swine flu), severe acute respiratory syndrome coronavirus (SARS-CoV), Middle East respiratory syndrome coronavirus (MERS-CoV), Ebola, Zika, SARS-CoV-2, and monkeypox virus (MXPV). The 2022 MPXV outbreak served as a stark reminder of global vulnerability, rapidly spreading to over 100 countries and prompting the World Health Organization (WHO) to declare a PHEIC on July 23, 2022.^1^ In 2024, WHO declared another PHEIC due to the resurgence of MPXV,^2^ particularly clade Ib, which has become endemic in the Democratic Republic of Congo (DRC).^3^ Sporadic cases have also been reported in Sweden, Thailand, and Germany.

MPXV was first identified in Denmark in 1958 and has been recognised as a zoonotic virus for decades; the first human case was reported in the DRC in 1970.^4^ While its natural reservoir remains unknown, MPXV can infect non-human primates and rodent species, such as squirrels and Gambian pouched rats.^5^ MPXV has recently been subdivided into four clades: Ia, Ib, IIa, and IIb.^3^ Mpox symptoms primarily include skin rashes, fever, swollen lymph nodes, and mucosal lesions.^6^ Mpox is endemic to Central and West Africa, with over 28,000 cases reported in the DRC and Nigeria between 2000 and 2019.^7^ The first mpox outbreak outside Africa occurred in 2003,^8^ with sporadic cases reported in the United Kingdom, Israel, and Singapore from 2018 to 2021.^9^, ^10^, ^11^ However, the ongoing mpox outbreak, driven by clade IIb, has spread to over 110 countries, resulting in more than 90,000 cases and 167 deaths by October 2023.^12^ In addition, in 2023, over 12,000 clade I infectious cases have been reported in the DRC, with a 4.6% mortality rate (581 deaths), primarily due to severe symptoms associated with clade I.^1^ Between late 2023 and early 2024, a new MPXV clade I lineage, Ib, was identified in Kamituga, DRC.^3^

Vaccination is crucial for controlling poxvirus outbreaks, particularly smallpox. Vaccinia virus (VACV) serves as the vaccine strain against poxviruses. First-generation smallpox vaccines, such as the Dryvax and Lister, were derived from calf lymph and played a key role in smallpox eradication.^13^ The second-generation ACAM2000 vaccine, developed using cell culture technology, is stockpiled for bioterrorism preparedness.^14^ Third-generation live-attenuated smallpox vaccines, including the Modified Vaccinia Ankara-Bavarian Nordic (MVA-BN)^15^ and LC16m8,^16^ have demonstrated immunogenicity with tolerable safety in clinical trials. However, they were approved after smallpox eradication. The discontinuation of routine smallpox vaccination has left younger generations susceptible to orthopoxvirus infections. The rapid spread of clade Ib highlights the urgent need for updated vaccines to prevent future outbreaks. Currently, two vaccines are approved for mpox: Bavaria-Nordic MVA-BN (JYNNEOS)^17^ and the ACAM2000 vaccine.^18^

LC16m8 is an attenuated smallpox vaccine derived from the Lister strain using primary rabbit kidney (PRK) cells at low temperatures. After the 36th passage of the virus, 50 clones were selected, and 25 were evaluated for their growth in Vero cells. The clone with the lowest growth rate was named LC16 (Lister Clone 16). Following six additional passages in PRK cells, a single plaque was isolated as LC16m0. After three more passages, LC16m8 was selected for its reduced growth ability in Vero and PRK cells.^19^ Compared to LC16m0, LC16m8 carries a single nucleotide deletion in B5, contributing to its attenuation.^20^ In Japan, LC16m8 was approved for smallpox in 1975 and mpox in 2022. Recent phase I clinical trials have demonstrated that LC16m8 vaccination induces neutralising antibodies against LC16m8, Zr599 (clade Ia), and Liberia (Clade IIa) strains without serious adverse effects.^21^

MPXV, a double-stranded DNA virus with a genome of approximately 197 kb, encodes around 180 proteins and exists in two infectious forms: intracellular mature and extracellular enveloped virions.^22^ Intracellular mature virions lack the additional outer membrane present in extracellular enveloped virions, making it challenging to identify the most effective antigen. However, single-antigen immunisation can confer protection against VACV and MPXV infections.^23^^,^^24^ Moreover, several studies have demonstrated that combining multiple antigens and antibody cocktails enhances protective effects.^24^, ^25^, ^26^, ^27^

Although LC16m8 has demonstrated immunogenicity and efficacy in preclinical and clinical settings, further immunological and pathological analyses of LC16m8 in preclinical studies are essential to fully characterize its properties and to develop new vaccines. This study evaluated the immunogenicity of LC16m8 in three mouse strains and human specimens and conducted pathological analysis using a non-human primate model.

## Methods

### Ethics statement for mice

C57BL/6J and BALB/c mice were purchased from NIHON CLEA. All animal experiments were conducted following the institutional guidelines of the Institute of Medical Science, University of Tokyo (PA22-18) and National Institute of Infectious Diseases (122161, 122162-II, 122,163). CAST/EiJ mice were purchased from Jackson Laboratory. All experiments using CAST/EiJ mice were approved by the Tokyo Metropolitan Institute of Medical Science Animal Experiment Committee (approval number: 24-072) and conducted in accordance with the animal experimentation guidelines of the Tokyo Metropolitan Institute of Medical Science. No mice were excluded based on predefined criteria. Randomisation was performed only before treatment.

### Ethical statement for non-human primate

The animals were housed in a biosafety level 2 facility at the Tsukuba Primate Research Centre (TPRC) of the National Institutes of Biomedical Innovation, Health, and Nutrition (NIBIOHN). The studies were conducted at the TPRC, NIBIOHN, following approval from the Committee on the Ethics of Animal Experiments of NIBIOHN, in accordance with its animal experiment guidelines (DSR04-33R1). The animals were cared for under the supervision of veterinarians in charge of the facility.

### Proteins

Sequences encoding extracellular region of the following envelope proteins (H3, Uniprot Q1M2A5, residues 2-244; L1, Uniprot Q76RD2, residues 6-182; D8, Uniprot O57211, residues 2-261; and A38, Uniprot A0A2I2MC98, residues 14-116) were amplified from the genomic DNA of VACV (strain Acanbis) and cloned into pcDNA3.4 vector (Thermo Fisher) carrying an artificial signal sequence and C-terminal human IgG1 Fc. All proteins were expressed as Fc fusion proteins using the Expi293F Expression System (Thermo Fisher Scientific) and were purified by affinity chromatography using rProtein A Sepharose Fast Flow (Cytiva). The Fc tag was removed using IdeS protease treatment, followed by protein A chromatography, as described previously.^28^ To study antibody responses in human samples, VACV A27 protein was synthesised using a wheat cell-free protein production method (WEPRO7240H, CellFree Sciences) and purified by affinity chromatography using Ni Sepharos Fast Flow (Cytiva). All other envelope proteins, including VACV proteins A27 and B5, and MPXV (clade Ia, Zaire) proteins H3, A35, I1, M1, L1, A29, and B6, were purchased from Sino Biological. MPXV (clade Ib, Congo, GenBank: PP601222.1) protein A35, residues 60-182, was expressed in-house as a C-terminal His-tagged protein.

To study different administration routes in mice, the human codon-optimised nucleotide sequence encoding the extracellular regions of the envelope proteins (L1, A33, and D8) from LC16m8 (GenBank accession no. AY678275.1), were synthesised (Eurofins Genomics). The sequences included histidine and Avi-tags. Recombinant proteins were produced using Expi293F cells, according to the manufacturer's instructions (Thermo Fisher Scientific). For biotinylation, the BirA expression vector was cotransfected, and biotin was supplemented at a concentration of 100 μM in the culture medium. Supernatants from the transfected cells were harvested five days post-transfection, and recombinant proteins were purified using Ni–nitrilotriacetic acid (Ni-NTA) agarose (QIAGEN).

### Viruses

The LC16m8 strain was obtained from Drs. Shimojima and Ebihara of the National Institute of Infectious Diseases (NIID). The virus was propagated in Vero cells and stored at −80 °C. Viral titres were determined using a plaque assay. Of the three MPXV strains (Zr-599; Congo Basin strain, clade I, Liberia; West African strain, clade IIa, and TKY220091; MPXV/human/Japan/Tokyo/TKY220091/2022, clade IIb; GenBank accession No. LC722946.1),^29^ Zr-599, and Liberia strains were kindly provided by the NIID, and the TKY220091 strain was kindly provided by the Tokyo Metropolitan Institute of Public Health.

### Immunisation

Six-to eight-week-old C57BL/6J and BALB/c mice were immunised with either phosphate-buffered saline (PBS) or LC16m8 (1 × 10^5^ plaque forming units [pfu]) at the base of the tail on day 0. Forty-seven days after immunisation, blood and inguinal lymph nodes were collected, and plasma and single-cell suspensions were prepared. The spleen was collected 21 days post-immunisation, and single-cell suspensions were generated to assess T-cell responses. In some experiments, mice were immunised subcutaneously or intraperitoneally with LC16m8 (2 × 10^6^ pfu), and blood samples were collected. To study different administration routes in mice, blood was collected 21 days post-administration.

### Enzyme-linked immunosorbent assay (ELISA) for antibody responses

Antigen-specific plasma antibody titres were measured using ELISA. Briefly, flat bottom, high-binding 96-well plates were coated with VACV or MPXV protein (1 μg/mL) in bicarbonate buffer and incubated overnight at 4 °C. The plates were then blocked with PBS (Nacalai Tesque) containing 1% BSA (SIGMA) for 60 min at room temperature. After washing three times with PBST (0.05% Tween 20), the plates were incubated with the diluted plasma for 2 h at room temperature. Following another three washes with PBST, the plates were incubated with horseradish peroxidase (HRP)-labelled goat anti-mouse total IgG antibody (Southern Biotech, RRID: AB_2619742) at room temperature for 90 min. For human samples, after three PBST washes, the plates were incubated with HRP-conjugated AffiniPure goat anti-human IgG (H + L) antibody (Proteintech, RRID: AB_2890979) for 90 min at room temperature. For non-human primate samples, the plates were incubated under the same conditions with HRP-labelled goat anti-monkey IgG antibody (Nordic-MUbio, cat#: GAMon/IgG (H + L)/PO). After three additional PBST washes, TMB peroxidase substrate buffer (KPL) was added, and the plates were incubated at room temperature for 10 min. The reaction was then stopped by adding 1N H_2_SO_4_ (Nacalai Tesque). The optical density (OD) at 450 and 540 nm was measured using a spectrophotometer. The reciprocal plasma dilution at which OD_450_-OD_540_ reached 0.3 for mice or 0.2 for humans, was defined as the antigen-specific plasma total IgG titre.

To study different administration routes in mice, ELISA plates were coated with each protein at 2 μg/mL. After blocking with PBS containing 1% BSA, serially diluted sera or monoclonal antibodies were applied to the plates and incubated with goat anti-mouse IgG1-HRP (Southern Biotech, RRID: AB_2794426), IgG2a-HRP (Southern Biotech, RRID: AB_2736843), or IgG2c-HRP (Southern Biotech, RRID: AB_2794462). HRP activity was visualised using an OPD substrate (Sigma–Aldrich), and OD_490_ was measured with an iMark Microplate Reader (BioRad). The following monoclonal antibodies were used to generate a calibration curve for quantifying the antibody concentration (L1, M2E9, D8, JF11, A33, and 6C).^30^, ^31^, ^32^

### Generation of monoclonal antibodies for ELISA

Recombinant monoclonal antibodies (mAbs) were prepared as previously described.^33^^,^^34^ The monoclonal antibodies were generated based on those previously reported as follows: anti-B5 mAb,^35^ anti-A27 mAb,^23^ anti-A33 mAb,^32^ anti-H3 mAb,^36^ anti-D8 mAb,^31^ and anti-L1 mAb.^30^ Briefly, the VH/VL genes of each clone were inserted into expression vectors for mouse IgG1, IgG2a, or IgG2c heavy chain, and kappa or lambda light chain. Expression plasmids encoding the heavy and light chains of Fab fragments were also constructed. Heavy and light chain expression vector pairs were transfected into Expi293F cells using the ExpiFectamine reagent (Thermo Fisher Scientific) according to the manufacturer's instructions. The supernatant was collected four days post-transfection. IgG and Fab antibodies were purified from the culture supernatant using a protein G column (Thermo Fisher Scientific) and Talon resin affinity chromatography (Clontech), respectively, and then dialysis against PBS before further analysis. Size-exclusion chromatography was performed to remove soluble aggregates for subsequent experiments. After centrifugation, the supernatant containing IgG1, IgG2a, or IgG2c was loaded onto a Superdex 200 10/300 GL column (Cytiva) at a 0.5 mL/min flow rate using PBS as the running buffer. A gel Filtration Standard (BioRad) was used according to the manufacturer's instructions. Each antibody was confirmed by ELISA to bind specifically and in a concentration-dependent manner to the recombinant proteins produced as described in the “Proteins” section.

### Flow cytometry analysis for Germinal Centre B (GC B) cells and T follicular helper (T_FH_) cells

A single-cell suspension of the inguinal lymph nodes was stained with LIVE/DEAD Aqua (Thermo Fisher Scientific) to identify dead cells. The cells were then washed and stained with the following antibodies from BioLegend: anti-CD3e (clone: 145-2C11, RRID: AB_1877170), anti-CD19 (clone: 6D5, RRID: AB_2564001), anti-CD38 (clone: 90, RRID: AB_312933), anti-GL7 (clone: GL7, RRID: AB_2563285), anti-CD4 (clone: RM4-5, RRID: AB_493701), anti-CD8a (clone: 53–6.7, RRID: AB_11204079), anti-CD185 (clone: L138D7, RRID: AB_2734207), and anti-PD-1 (clone: 29F.1A12, RRID: AB_1877231) antibodies. GC B and T_FH_ cells were analysed using flow cytometry.

### Splenocyte stimulation

Single-cell suspensions of splenocytes were stimulated with medium or LC16m8 for 24 h. Interferon (IFN)-γ and Interleukin (IL)-13 concentrations in supernatants were measured by ELISA kit (R&D).

### Immunisation and viral challenge in CAST/EiJ mice

Thirteen-to fourteen-weeks-old CAST/EiJ mice were immunised with either vehicle or LC16m8 (1 × 10^5^ pfu) at the base of the tail on day 0. Twenty-eight days post-immunisation, the mice were inoculated intranasally with 1 × 10^4^ pfu of MPXV Zr-599. Body weight was monitored daily, and the mice were euthanised nine days post infection to collect lung samples. To assess viral replication, serial dilutions of the supernatant acquired from the mouse left lung lobe homogenate (5% w/v in HBSS) were inoculated into confluent VeroE6 cells in a 6-well plate and incubated for 1 h. The samples were then removed, and VeroE6 cells were cultured in MEM supplemented with 10% inactivated foetal bovine serum (FBS) for 3 days. The plates were fixed with 10% buffered formalin, and plaques were visualised by crystal violet staining.

### Preparation and stimulation of human peripheral blood mononuclear cells (PBMCs)

Plasma and PBMCs were obtained from six healthy adult male volunteers before and one month post-LC16m8 vaccination, which is licenced for human use. All immunological experiments involving human PBMCs before and after vaccination were approved by the Institutional Review Board of the Institute of Medical Science, University of Tokyo (2021-8-0520). All participants provided signed informed consent prior to participation. After preparation of PBMCs using Ficoll (Cytiva), the cells and plasma were stored in Cell Banker (Zenogen Pharma) in a liquid nitrogen tank.

### Flow cytometry analysis for activation-induced marker (AIM) assay

After thawing, PBMCs were plated at a density of 8 × 10^5^ cells/mL and maintained in complete RPMI (RPMI 1640 medium supplemented with 10% FCS, penicillin, and streptomycin). The cells were then stimulated with medium, LC16m8, or SARS-CoV-2 spike peptide pool for 24 h. Following stimulation, the cells were stained with LIVE/DEAD Far Red to identify dead cells. After washing, cells were then stained with anti-CD3 (BioLegend; clone: SK7, RRID: AB_2563420), anti-CD19 (BioLegend; clone: HIB19, RRID: AB_314248), anti-CD14 (BioLegend; clone: M5E2, RRID: AB_493695), anti-CD56 (BioLegend; clone: HCD56, RRID: AB_10896424), anti-CD4 (BioLegend; clone: OKT4, RRID: AB_1186122), anti-CD8a (BioLegend; clone: RPA-T8, RRID: AB_2563264), anti-CD137 (BioLegend; clone: 4B4-1, RRID: AB_2207741), anti-CD134 (BioLegend; clone: Ber-ACT35, RRID: AB_10719958), and anti-CD69 (BD Pharmingen; clone: FN50, RRID: AB_395916) antibodies. Activated CD4^+^ and CD8^+^ T cells were analysed using flow cytometry.

### ELISPOT assay

After thawing, PBMCs were plated at a density of 4 × 10^5^ cells/mL and maintained in complete RPMI (RPMI 1640 medium supplemented with 10% FCS, penicillin, and streptomycin). The cells were stimulated with the medium, LC16m8, or SARS-CoV-2 spike peptide pool along with an anti-CD28 antibody (BioLegend; clone: CD28.2, RRID: AB_2616667) (2 μg/mL) for 24 h. Following stimulation, ELISPOT assays were performed using Human IFN-g/IL-2 FluoroSpot (C.T.L.).

### Cytopathic effect endpoint neutralisation assay

Neutralisation of LC16m8 was assessed using a limiting dilution model.^37^ Heat-inactivated human plasma was serially diluted (2-, 4-, 8-, 16-, 32-, 64-, and 128-fold) in Eagle's minimum essential medium (EMEM) supplemented with 2% FBS, penicillin, and streptomycin. The plasma dilutions were then mixed with LC16m8 at a 1:1 ratio in a total volume of 16 μL and incubated at 37 °C for 1 h. Subsequently, 32 μL of EMEM with 2% FBS was added, and the 48 μL plasma-virus mixture was incubated at 4 °C overnight. The next day, 444 μL EMEM with 2% FBS was mixed with 36 μL of the incubated plasma-virus mixture. Vero E6 cells were pre-seeded in flat bottom 96-well plates at 2 × 10^4^ cells/well. The culture medium (100 μL/well) was then replaced with an equal volume of the incubated mixture in quadruplicate. The inoculated plates were incubated at 37 °C with 5% CO_2_ for 48–72 h, and the cells were fixed with 0.5 mL/well of 4% paraformaldehyde. After air drying at room temperature for at least 3 h, the cells were stained with 2% crystal violet in 20% ethanol. Cytopathic effect was determined based on whether cells detachment exceeded 50% per well, and the 50% neutralisation titre (NT_50_) was calculated using the Spearman–Karber method. Data from at least two independent experiments were summarised.

### Plaque reduction neutralisation titre (PRNT) assay

Neutralisation assays against mpox viruses were performed as described previously.^21^ Briefly, heat-inactivated human plasma was diluted as described above in Dulbecco's Modified Eagle's Medium supplemented with 2% FBS, penicillin, and streptomycin. The diluted plasma was mixed with MPXV_Liberia, MPXV_Zr599, and MPXV_TKY220091 at a 1:1 ratio in a total volume of 100 μL. RK13 cells were seeded in 24-well plates at a density of 2 × 10^5^ cells/mL. After 18 h of incubation at 37 °C in a humidified atmosphere of 5% CO_2_, the plasma-virus mixture was added to the pre-seeded RK13 cells along with 0.3 mL/well of fresh Dulbecco's Modified Eagle's Medium containing 5% FBS and Pen-Strep. Following incubation for 72–96 h, the cells were fixed with 1 mL/well of 10% neutral-buffered formalin or 4% paraformaldehyde, dried, and stained with crystal violet. Plaques were counted using the Image J macro language, ViralCounter_0.7, with minor modifications, and manually confirmed. Summarised data were obtained from the average of at least two independent experiments. All the MPXV-related experiments were conducted in a BSL-3 facility at the Institute of Medical Science, University of Tokyo.

### LC16m8 immunisation in cynomolgus monkey

Three cynomolgus monkeys were intradermally (i.d.) immunised with the LC16m8 strain (10^8^ pfu) on day 0. Blood samples were collected on days 0 and 56.

### LC16m8 challenge in cynomolgus monkey

Four adult cynomolgus macaques, all confirmed negative for simian immunodeficiency virus, simian type D retrovirus, simian T-cell lymphotropic virus, simian foamy virus, Epstein–Barr virus, cytomegalovirus, and B virus, were used in this study. All cynomolgus macaques were intravenously inoculated with 10^8^ pfu of LC16m8. Animal procedures were performed under anaesthesia. Blood samples were collected at designated time points. The body conditions, including temperature and weight, were monitored throughout the study. White blood cell (WBC) and red blood cell (RBC) counts were determined using an automatic blood cell analyser (Sysmex). Of the four animals, two were euthanised 10 days post infection (endpoint 1), while the remaining two were euthanised 20 days post-infection (endpoint 2) (Fig. 6a).

### Purification of DNA and quantitative PCR (qPCR) analysis

The amount of viral DNA was quantified during necropsy. Tissues and organs samples were collected using a biopsy punch instrument (5 mm; Kaijirushi) and preserved in a DNAlater solution (Invitrogen). A weight-scaled portion of each sample was used for DNA extraction. Tissue samples were homogenised using MagNA Lyser Green Beads with a MagNA Lyser, and DNA was extracted using a DNeasy Blood & Tissue kit (QIAGEN) according to the manufacturer's instructions. The extracted DNA was then subjected to qPCR using the orthopoxvirus qPCR Test Kit (Youseq). The qPCR reaction was run on a StepOne Real-Time PCR System (Applied Biosystems). The reaction conditions were 95 °C for 3 min, and 45 cycles of 15 s at 95 °C followed by 60 s at 60 °C.

### Serum biochemical analysis

Whole blood was collected in a blood collection tube (plain, 5 mL) and allowed to stand at room temperature for at least 30 min before centrifugation at 1200×*g* for 10 min. Serum C-reactive protein (CRP) and creatine phosphokinase (CPK) levels were measured using a calibrated Fuji DRI-CHEM system (FUJIFILM, Tokyo, Japan).

### Cytokine and chemokine profiles

Serum cytokine and chemokine profiles were analysed using a Cytokine Monkey Magnetic 29-Plex Panel on the Luminex platform (Invitrogen, Thermo Fisher Scientific) following the manufacturer's protocol. Beads were washed with 2% paraformaldehyde solution before measurement, and signals were then detected and analysed using a Luminex Bio-Plex System (Luminex) with Bio-Plex Manager 5.0 software (Luminex).

### Flow cytometry analysis for monkey PBMCs

To analyse the myeloid cell population, PBMCs were stained with LIVE/DEAD Far Red (Thermo Fisher Scientific) to identify dead cells. The cells were then stained with anti-CD45 (BD; clone: D058-1283, RRID: AB_2871344), anti-CD86 (BD; clone: 2331, RRID: AB_2744453), anti-CD11c (BD; clone: SHCL-3, RRID: AB_2742232), anti-HLA-DR (BD; clone: G46-6, RRID: AB_2738558), anti-CD14 (BD; clone: M5E2, RRID: AB_10611732), anti-CD16 (BioLegend, clone: 3G8, RRID: AB_2563801), and anti-CD3 (Miltenyi; clone: 10D12, RRID: AB_871672) antibodies. For anti-CD3 staining, BUV737 streptavidin (BD, RRID: AB_2870104) was used, and CD3 was included as a lineage marker. Following fixation and permeabilization, cells were stained with anti-IFN-γ (BD; clone: 4S.B3, RRID: AB_2738952), anti-TNF-α (BioLegend; clone: Mab11, RRID: AB_2565858), and IL-6 (BD; clone: MQ2-6A3, RRID: AB_395515) antibodies. Myeloid cell populations and cytokine-producing myeloid cells were analysed using flow cytometry.

To analyse the T-cell population, the cells were stained with LIVE/DEAD Far Red to identify dead cells. The cells were then stained with anti-CD45 (BD; clone: D058-1283, RRID: AB_2871344), anti-CD3 (BD; clone: SP34-2, RRID: AB_2738603), anti-CD4 (BD; clone: OKT4, RRID: AB_2875058), anti-CD8 (BioLegend; clone: SK1, RRID: AB_2044008), anti-CD20 (BioLegend; clone: 2H7, RRID: AB_2565524), anti-CD11b (Miltenyi; clone: M1/70.15.11.5, RRID: AB_2726044), anti-CD14 (Miltenyi; clone: TUK4, RRID: AB_2725973), anti-CD33 (Miltenyi; clone: AC104.3E3, RRID: AB_2726122), and anti-CD66abce (Miltenyi; clone: TET2, RRID: AB_2751886) antibodies. Anti-CD20, anti-CD11b, anti-CD14, anti-CD33, and anti-CD66abce antibodies were used as lineage markers, with BB515 streptavidin (BD Biosciences, RRID: AB_2869580) for lineage marker staining. Following fixation and permeabilization, cells were stained with anti-IFN-γ (BD; clone: 4S.B3, RRID: AB_2738952), anti-TNF-α (BioLegend; clone: Mab11, RRID: AB_2565858), and IL-6 (BD; clone: MQ2-6A3, RRID: AB_395515) antibodies. T-cell populations and cytokine-producing T cells were analysed using flow cytometry.

### Statistical analysis

Statistical significance was assessed using the Mann–Whitney U test, Kruskal–Wallis test followed by Dunn's multiple comparison test, or signed rank test (binomial test). Shapiro–Wilk and Levene's tests were performed for normality and homogeneity of variance, respectively. For the cynomolgus monkey dataset (days 0–10), the area under the curve (AUC) was used as a summary measure. AUC values were calculated for each monkey, followed by statistical analysis using the Mann–Whitney U test. All statistical analyses were performed using GraphPad Prism 10 (GraphPad, Inc.) and RStudio.

### Role of funders

The funders had no role in the study design, data analysis, data interpretation, manuscript writing, or any other aspect of this study.

## Results

### Immunological analysis of LC16m8 in mouse

**Fig. 1:**
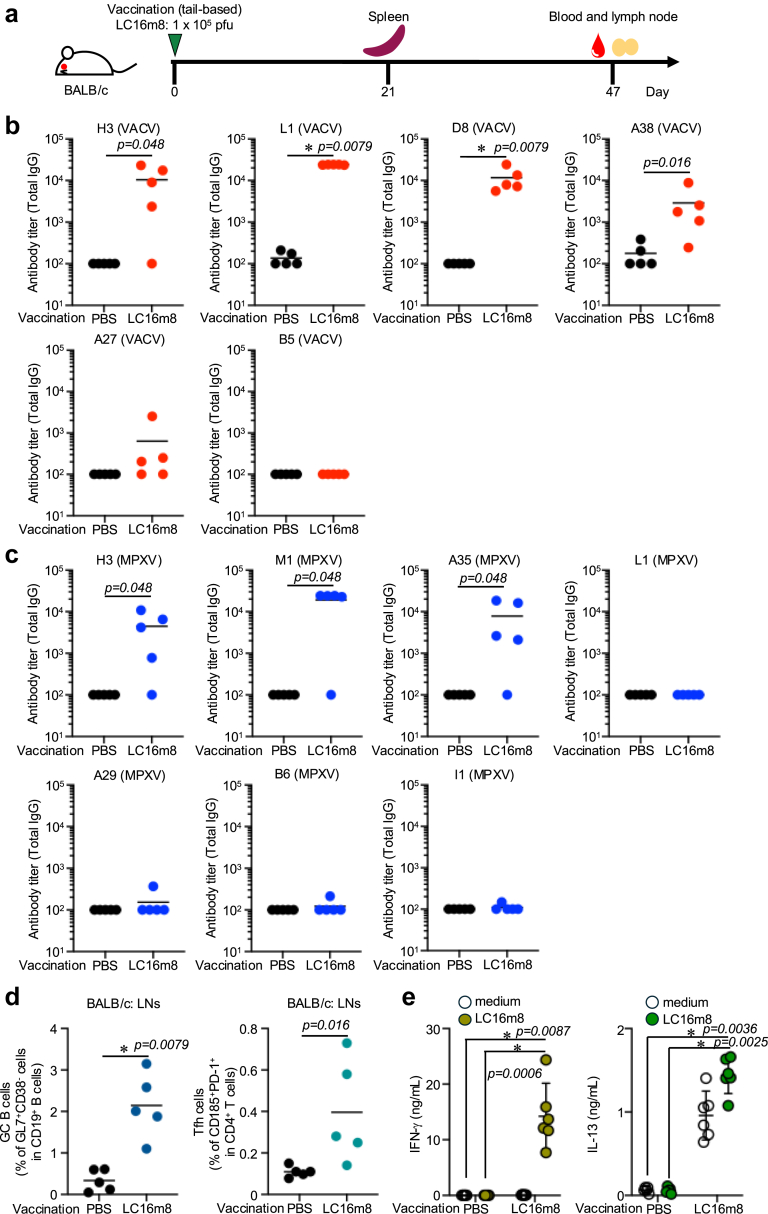
**Immunogenicity of LC16m8 strain in BALB/c mice**. (a) Schedule of LC16m8 strain vaccination and sample collection. Six-to eight-weeks old female BALB/c mice (n = 5) were immunised at the tail base with either PBS or the LC16m8 strain (1 × 10^5^ pfu) at day 0. Blood samples and inguinal lymph nodes were collected 47 days post-vaccination. For T-cell experiments, spleens were collected 21 days after vaccination from PBS-immunised mice (n = 5) and LC16m8-immunised mice (n = 6). (b) Total IgG antibody titres against H3, L1, D8, A38, A27, and B5 of VACV as measured by ELISA 47 days post-immunisation. (c) Total IgG antibody titres against H3, L1, M1, A35, I1, A29, and B6 of MPXV as measured by ELISA 47 days post-immunisation. (d) The percentage of GC B and T_FH_ cells in the inguinal lymph nodes as analysed by flow cytometry 47 days post-immunisation. (e) IFN-γ and IL-13 concentration in LC16m8 stimulated splenocyte culture supernatant as measured by ELISA. (b–d) Data are presented as mean values. (e) Data are shown as mean values with 95% confidence intervals. Statistical significance was assessed using the Mann–Whitney U test (b–d) and the Kruskal–Wallis test followed by Dunn's multiple comparisons test (e). (E left: IFN- γ) p-value of PBS immunisation medium restimulation vs. LC16m8 immunisation LC16m8 restimulation is 0.0087, PBS immunisation LC16m8 restimulation vs. LC16m8 immunisation LC16m8 restimulation is 0.0006. (e right: IL-13) p-value of PBS immunisation medium restimulation vs. LC16m8 immunisation LC16m8 restimulation is 0.0036, PBS immunisation LC16m8 restimulation vs. LC16m8 immunisation LC16m8 restimulation is 0.0025.

**Fig. 2:**
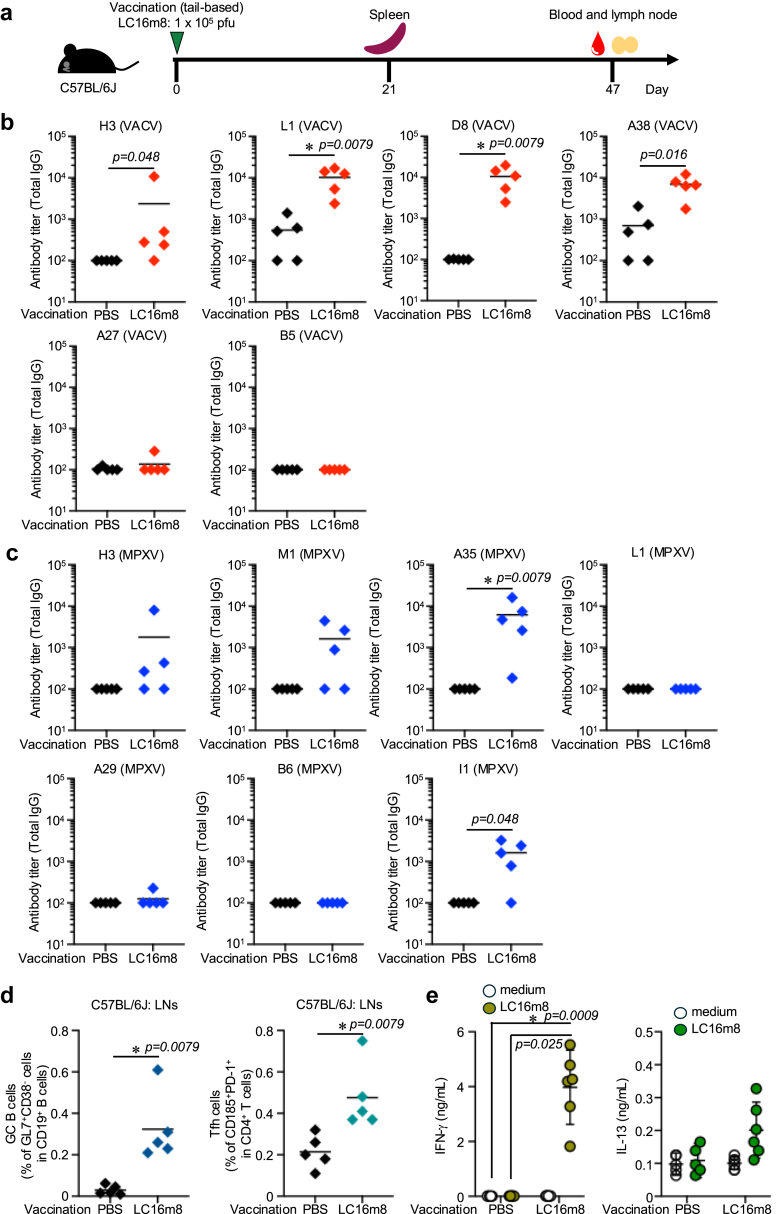
**Immunogenicity of LC16m8 strain in C57BL/6J mice**. (a) Schedule of LC16m8 strain vaccination and sample collection. Six-to eight-weeks old female C57BL/6J mice (n = 5) were immunised at the tail base with either PBS or the LC16m8 strain (1 × 10^5^ pfu) at day 0. Blood samples and inguinal lymph nodes were collected 47 days post-vaccination. For T-cell experiments, spleens were collected 21 days after vaccination from PBS-immunised mice (n = 5) and LC16m8-immunised mice (n = 6). (b) Total IgG antibody titre against H3, L1, D8, A38, A27, and B5 of VACV as measured by ELISA 47 days post-immunisation. (c) Total IgG antibody titre against H3, L1, M1, A35, I1, A29, and B6 of MPXV as measured by ELISA 47 days post-immunisation. (d) The percentage of GC B and T_FH_ cells in the inguinal lymph nodes as analysed by flow cytometry 47 days post-immunisation. (E) IFN-γ and IL-13 concentration in LC16m8-stimulated splenocyte culture supernatant as measured by ELISA 21 days post-immunisation (b–d) Data are shown as mean values. (e) Data are shown as mean values with 95% confidence intervals. Statistical significance was assessed using the Mann–Whitney U test (b–d) and Kruskal–Wallis test followed by Dunn's multiple comparisons test (e). (e left: IFN- γ) p-value of PBS immunisation medium restimulation vs. LC16m8 immunisation LC16m8 restimulation is 0.0009, PBS immunisation LC16m8 restimulation vs. LC16m8 immunisation LC16m8 restimulation is 0.025.

First, we evaluated the immunogenicity of LC16m8 in two different mouse strains: BALB/c and C57BL/6J. To assess the antibody responses induced by LC16m8 immunisation, we used several proteins: H3, L1, D8, A38, A27, and B5 from VACV, and H3, L1, M1, A35, I1, A29, and B6 from MPXV. Among these, A27 and A29, B5 and B6, and L1 and M1 are the orthologues of VACV and MPXV, respectively (Table 1). Mice received a single tail-based immunisation of LC16m8, and antibody responses were induced against most VACV-derived proteins, except for A27 and B5 in BALB/c mice (Fig. 1b). For MPXV proteins, antibody responses were observed only for H3 (p = 0.048 by Mann–Whitney U test), M1 (p = 0.048 by Mann–Whitney U test), and A35 (p = 0.048 by Mann–Whitney U test) but not for I1, A29, or B6 (Fig. 1c). The absence of antibody responses to B5 and B6 is consistent with previous reports, as B5 is not expressed in LC16m8.^38^ Next, we measured the induction of GC B and T_FH_ cells in draining lymph nodes. Forty-seven days post-immunisation, the mice showed a high frequency of GC B and T_FH_ cells (Fig. 1d: p = 0.0079 for GC B cells, p = 0.016 for T_FH_ cells by Mann–Whitney U test). Similar results were observed in C57BL/6J mice, although antibody responses and GC B cell induction were lower than in BALB/c mice, except for antibody responses against the I1 protein (Fig. 2b–d).

**Table 1:** Amino acid sequence identity with VACV (Copenhagen) and LC16m8.

MPXV protein (NC_003310.1)	VACV protein (M35027.1)	VACV identity (%)	LC16m8 (AY678275.1)	LC16m8 identity (%)
A29 (110aa)	A27 (110aa)	103/110 (93.6%)	Cell fusion protein	105/110 (95.5%)
A35 (181aa)	A33 (185aa)	174/185 (94.1%)	EEV glycoprotein	174/185 (94.1%)
A40 (277aa)	A38 (277aa)	266/277 (96.03%)	CD47 antigen/integrin-associated protein	267/277 (96.39%)
B6 (317aa)	B5 (317aa)	304/317 (95.9%)	Deleted	
E8 (304aa)	D8 (304aa)	288/304 (94.7%)	Cell surface-binding protein	285/304 (93.8%)
H3 (324aa)	H3 (324aa)	304/324 (93.8%)	IMV membrane associated protein	304/324 (93.8%)
I1 (312aa)	I1 (312aa)	310/312 (99.4%)	Putative DBA-binding virion core protein	309/312 (99.04%)
L1 (152aa)	J1 (153aa)	147/153 (96.1%)	Dimeric virion protein	147/153 (96.1%)
M1 (247aa)	L1 (247aa)	247/250 (98.8%)	Myristylated membrane protein	247/250 (98.8%)

We also compared the effects of different immunisation routes for the LC16m8 strain in BALB/c and C57BL/6J mice. When evaluating antibody responses against A33, D8, and L1 of VACV, intraperitoneal immunisation with LC16m8 induced slightly higher antibody responses in BALB/c mice (Supplemental Figure S1a). In contrast, C57BL/6J mice showed comparable antibody responses regardless of the immunisation route (Supplemental Figure S1b). To further characterize the immune response, we measured IgG subclasses following a single LC16m8 immunisation. The levels of IgG2a/c against A33 in BALB/c and L1 and A33 in C57BL/6J mice were higher than those of IgG1 (Supplemental Figure S1a: p = 0.0077 by Kruskal–Wallis test followed by Dunn's multiple comparisons test; b: p = 0.016 for A33 s.c. route and p = 0.014 for L1 between the i.p. route by Kruskal–Wallis test followed by Dunn's multiple comparisons test). These results suggest that LC16m8 induces Th1-type adaptive immune responses in BALB/c and C57BL/6J mice.

Although CD4^+^ and CD8^+^ T cells are dispensable for protection against MPXV challenge,^39^ recent studies indicate that T-cell responses, especially Th1 memory CD4^+^ T cells, provide protective immunity against orthopoxviruses.^40^ To assess T-cell responses in mice, we stimulated splenocytes from LC16m8-vaccinated mice. LC16m8-immunised BALB/c mice showed a significant increase in IFN-γ production (Fig. 1e p = 0.0006 by Kruskal–Wallis test followed by Dunn's multiple comparisons test). Although the baseline IL-13 production in the LC16m8-immunised group was high, LC16m8 restimulation led to a slight increase in IL-13 production (Fig. 1e; p = 0.0025 by the Kruskal–Wallis test followed by Dunn's multiple comparisons test). Similar T-cell responses were observed in C57BL/6J mice (Fig. 2e). LC16m8 vaccination significantly induced LC16m8-specific IFN-γ-production (Fig. 2e; p = 0.025 by Kruskal–Wallis test followed by Dunn's multiple comparison test). These findings suggest that the LC16m8 strain induces Th1-type adaptive immune responses in both BALB/c and C57BL/6J mice. In addition, we utilised a peptide library of several VACV proteins, but none of the peptides induced T-cell responses following LC16m8 vaccination (data not shown).

### LC16m8 strain protects the mice from MPXV challenge

**Fig. 3:**
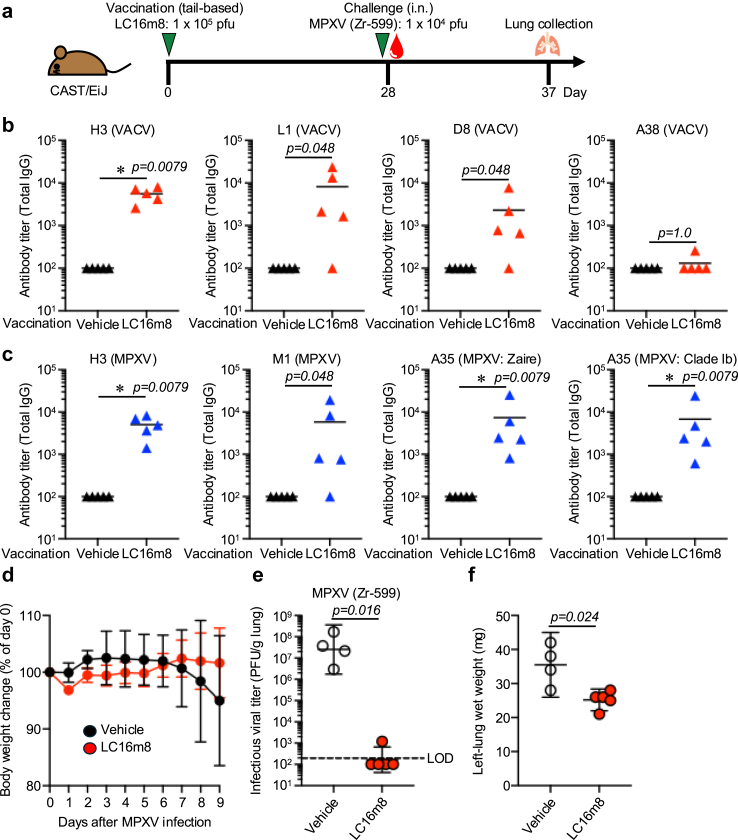
**LC16m8 strain protects the mice from MPXV challenge**. (a) Schedule of LC16m8 strain vaccination and sample collection. Thirteen-to fourteen-weeks-old CAST/EiJ mice (n = 5) immunised with either vehicle or LC16m8 (1 × 10^5^ pfu) at the tail base on day 0. Following blood sample collection 28 days post-vaccination, the mice were inoculated intranasally with 1 × 10^4^ pfu of the MPXV Zr-599 strain. Body weight was monitored daily. The mice were euthanised 9 days post-MPXV infection, and lung samples were collected from the vehicle-immunised (n = 4) and LC16m8-immunised (n = 5) mice. (b) Total IgG antibody titre against H3, L1, D8, and A38 of VACV were measured by ELISA 28 days post-immunisation. (c) Total IgG antibody titre against H3, M1, A35 (MPXV: Zaire), and A35 (MPXV: clade Ib) of MPXV was measured by ELISA 28 days post-immunisation. (d) Body weight was monitored. (e) Infectious viral titre in the lung was measured by plaque assay. (f) Lung wet weight was measured. (d–f) One mouse in the vehicle-immunised group died during anaesthesia for infection and was excluded from the analysis, as no data could be collected. (b–c) Data are shown as mean values. (d and f) Data are shown as mean values with 95% confidence intervals (e) Data are shown as geometric means with 95% confidence intervals. (b, c, e, and f) Statistical significance was assessed using the Mann–Whitney U test.

Next, we evaluated the efficacy of the LC16m8 strain using the CAST/EiJ strain, which is highly susceptible to MPXV infection and known to exhibit weight loss, morbidity, and mortality following a lethal dose challenge.^41^ To assess the antibody response induced by LC16m8 immunisation, we measured IgG titres against VACV and MPXV proteins. Similar to BALB/c and C57BL/6J mice, CAST/EiJ mice developed significant antibody responses against VACV proteins H3 (p = 0.0079 by Mann–Whitney U test), L1 (p = 0.048 by Mann–Whitney U test), and D8 (p = 0.048 by Mann–Whitney U test). However, no significant response was observed against A38 (p = 1.0, Mann–Whitney U test) (Fig. 3b). For MPXV proteins, antibody responses were also induced against H3 (p = 0.0079 by the Mann–Whitney U test), M1 (p = 0.048 by the Mann–Whitney U test), and A35 (p = 0.0079 by the Mann–Whitney U test). These findings are consistent with the responses observed in BALB/c and C57BL/6J mice (Fig. 3c). To investigate the effect of a mutation in the current clade Ib MXPV outbreak, we synthesised the A35 protein based on the clade Ib sequence. Since only a single mutation was identified in clade Ib, the antibody response against this variant remained comparable (p = 0.0079, Mann–Whitney U test).

To assess the protective efficacy of LC16m8 against MPXV, the CAST/EiJ mice were challenged with MPXV Zr-599 (1 × 10^4^ pfu) four weeks post-immunisation, and monitored for changes in body weight. Although both vehicle-treated and LC16m8-immunised mice showed similar levels of body weight loss (Fig. 3d), the viral load in the lungs was significantly lower in the LC16m8-immunised mice (Fig. 3e; p = 0.016 by the Mann–Whitney U test). Four out of five mice had viral loads below the limit of detection. The remaining mouse had a detectable viral load but significantly lower than the vehicle-treated group. Lung wet weight was measured as an indicator of pulmonary oedema, and LC16m8 immunisation reduced the wet weight of the left lung (Fig. 3f; p = 0.024, Mann–Whitney U test). These results demonstrate that LC16m8 exhibits potent efficacy against MPXV infection in a mouse model.

### Immunological analysis of LC16m8 in vaccinated humans

**Fig. 4:**
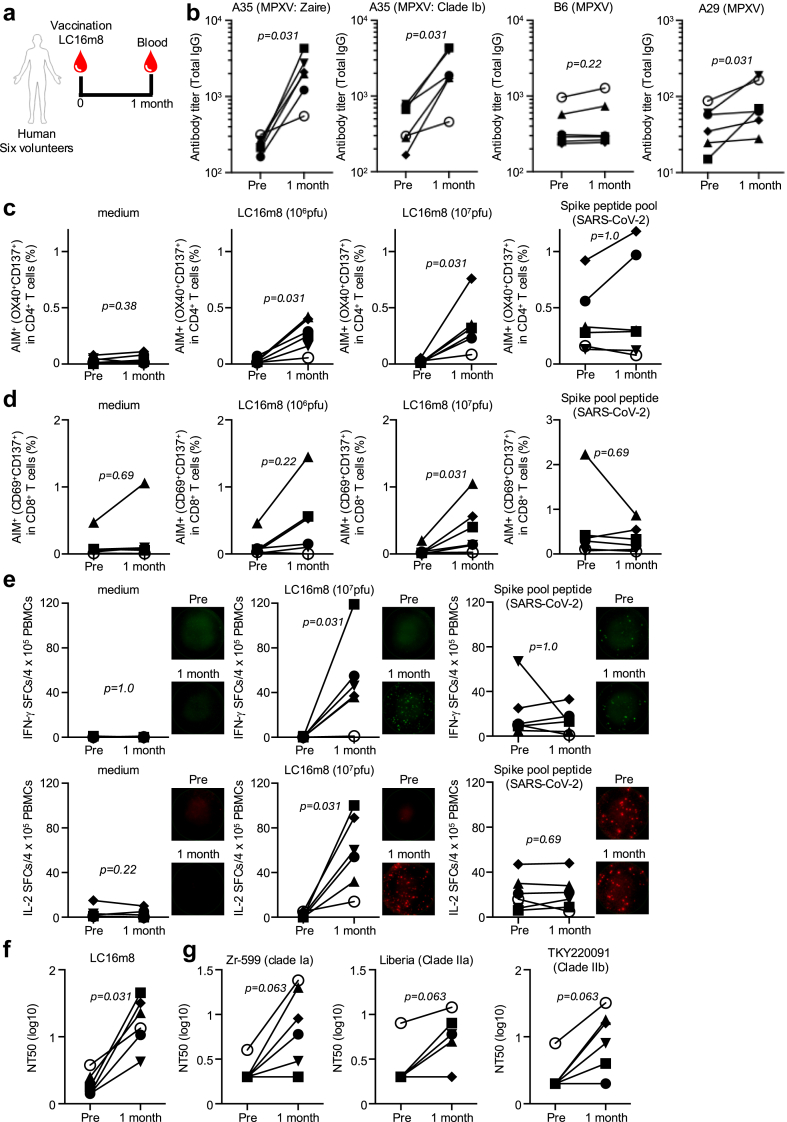
**Immunogenicity of LC16m8 vaccine in humans**. (a) Blood samples from six healthy volunteers collected before and one month after receiving the smallpox vaccine LC16m8, were separated into plasma and PBMCs. (b) Plasma total IgG antibody against A29 (MPXV), A35 (MPXV: Zaire), A35 (MPXV: Clade Ib), and B6 (MPXV) as measured by ELISA. (c and d) Human PBMCs were stimulated with either medium, LC16m8 (10^6^ pfu), LC16m8 (10^7^ pfu), or spike peptide pool for 24 h. T-cell activation as measured by flow cytometry; (c) activated CD4^+^ T cells (OX40^+^CD137^+^) or (d) CD8^+^ T cells (CD69^+^CD137^+^). (e) Human PBMCs were incubated with either medium, LC16m8 (10^6^ pfu), LC16m8 (10^7^ pfu), or spike peptide pool together with α-CD28 antibody for 24 h. IFN-γ- and IL-2-producing cells were measured by ELISPOT. Neutralising antibody titre against (f) VACV and (g) MPXV as measured using human plasma. Statistical significance was assessed using a binomial test by RStudio.

To assess the immunogenicity of LC16m8 in humans, we analysed antibody responses in recently vaccinated individuals one month post-vaccination. IgG titres were measured against VACV A27 and MPXV A29, A35, and B6. Consistent with mouse data, LC16m8 vaccination did not induce antibody responses against B6R, the MPXV orthologue of VACV B5R, which is deleted from LC16m8 (Fig. 4b and Supplemental Figure S4). Antibody responses against MPXV A35 were elevated in five of six donors (Fig. 4b: p = 0.031 by binomial test). In contrast, antibodies against A27L VACV and A29 MPXV showed slight increases after vaccination (Fig. 4b and Supplemental Figure S4: p = 0.031 by binomial test). To evaluate the effect of the mutation in the current clade Ib outbreak, the antibody response against clade Ib MPXV was compared with those against clade Ia of MPXV (Fig. 4b; p = 0.031 by the binomial test).

To evaluate T-cell responses, we performed an AIM assay by stimulating human PBMCs with LC16m8 or SARS-CoV-2 spike pool peptide for 24 h LC16m8-specific CD4^+^ T-cell activation was detected in most donors, whereas SARS-CoV-2 spike-specific CD4^+^ T-cell responses remained unchanged (Fig. 4c: p = 0.38 (medium), p = 0.031 (LC16m8; 10^6^ pfu), p = 0.031 (LC16m8; 10^7^ pfu), and p = 1 (spike peptide pool) by the binomial test). In addition, LC16m8-specific CD8^+^ T-cell activation was detected one-month post-vaccination (Fig. 4d: p = 0.69 (medium), p = 0.22 (LC16m8; 10^6^ pfu), p = 0.031 (LC16m8; 10^7^ pfu), p = 0.69 (Spike peptide pool), by the binomial test). The ELISPOT assay demonstrated a significant induction of IFN-γ- and IL-2 production in response to LC16m8 restimulation one-month post-vaccination, which was absent before vaccination (Fig. 4e: p = 0.031 (IFN-γ), p = 0.031 (IL-2) by the binomial test). However, T-cell activation and cytokine production were not observed when using peptide pools (data not shown).

The neutralising antibody titre is a crucial marker for protection against infectious diseases. The plaque assay showed that LC16m8 vaccination significantly induced the production of neutralising antibodies against the vaccinated LC16m8 strain (Fig. 4f; p = 0.031, binomial test). Consistent with a recent clinical trial demonstrating neutralising antibody induction against MPXV, five of six donors exhibited enhanced neutralising activity against Zr-599 (clade Ia), Liberia (Clade IIa), and TKY220091 (Clade IIb) of MPXV, following LC16m8 vaccination.

### Safety profile of LC16m8 in non-human primate

**Fig. 5:**
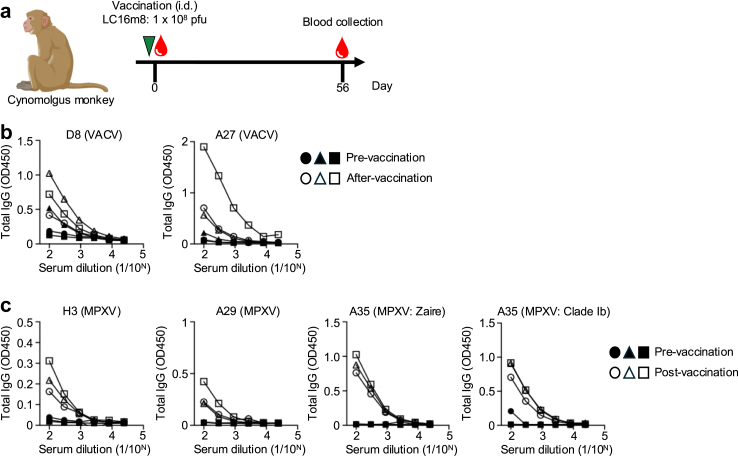
**Antibody responses by LC16m8 vaccination in non-human primate**. (a) Schedule of LC16m8 strain vaccination and sample collection. Cynomolgus monkeys (n = 3) were intradermally (i.d.) immunised with LC16m8 strain (10^8^ pfu) on day 0. Blood samples were collected on day 56. (b) Total IgG antibody response against D8 and A27 of VACV as measured by ELISA before vaccination and 56 days post-vaccination. (c) Total IgG antibody response against H3, A29, A35 (MPXV: Zaire), and A35 (MPXV: clade Ib) of MPXV as measured by ELISA before vaccination and 56 days post-vaccination.

Several studies have demonstrated the effectiveness of LC16m8 against lethal MPXV Zaire strains in non-human primate models.^42^^,^^43^ To further confirm the immunogenicity, three cynomolgus monkeys were intradermally immunised with LC16m8. Eight-weeks post-immunisation, robust antibody responses were observed against D8 and A27 of VACV (Fig. 5b) as well as H3, A35, and A29 of MPXV, including the A35 protein from the current outbreak strain (Fig. 5c). The efficacy of LC16m8 against MPXV Zaire strain infection was confirmed in these monkeys, which is consistent with previous findings (data not shown).

**Fig. 6:**
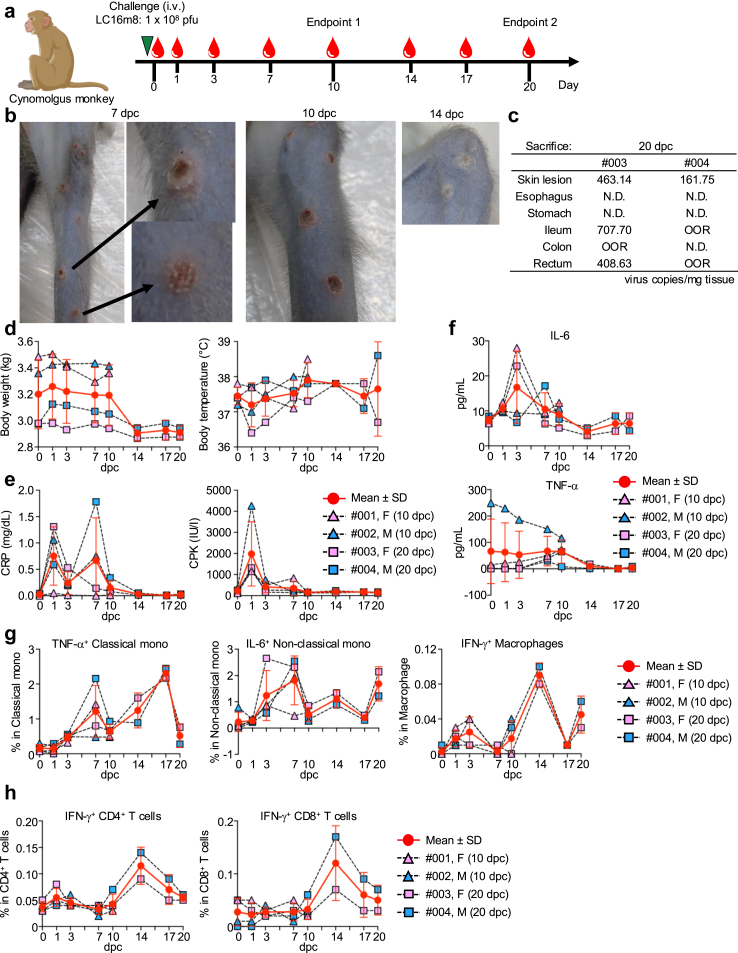
**The safety profile of the LC16m8 strain in non-human primates**. (a) Schedule of LC16m8 strain administration and sample collection. Two female and two male cynomolgus monkeys were intravenously (i.v.) challenged with LC16m8 strain (10^8^ pfu) on day 0. The blood samples were collected on days 0, 1, 3, 7, 10, 14, 17, and 20 post-viral challenge. Ten days and twenty days post–challenge, two cynomolgus monkeys were sacrificed for blood and tissue collection each (#001 and #002 for Endpoint 1, and #003 and #004 for Endpoint 2). (b) Pox lesions on the skin and tongue seven to ten days post-LC16m8 challenge. (c) Viral DNA copies in the skin lesion, oesophagus, stomach, ileum, colon, and rectum twenty days post–challenge harvested from two cynomolgus monkeys, as measured by q-PCR. (d) Changes in body weight and body temperature following LC16m8 strain challenge. (e) CRP and CPK levels in plasma as measured by the Fuji DRI-CHEM system. (f) IL-6 and TNF-α concentration in the plasma as measured by the Luminex platform. The percentage of (g) cytokine-producing myeloid cells and (h) Flow cytometry analysis of T-cells.

Finally, we used a non-human primate model to evaluate the safety profile of LC16m8. Four cynomolgus monkeys were intravenously challenged with a high dose of LC16m8 vaccine strain (1 × 10^8^ pfu). Two monkeys were sacrificed on days 10 and 20, designated as endpoints 1 and 2 (Fig. 6a). Blood samples were collected on days 0, 3, 7, 10, 14, 17 and 20. Pox lesions and skin scabs were monitored at each time point. Seven days post-viral challenge, pox lesions appeared randomly on the skin. Two weeks post–challenge, all skin lesions had scabbed, and no mortality was observed (Fig. 6b). To detect viral LC16m8 viral DNA in various organs, qPCR was performed on samples from lungs, spleen, kidneys, liver, skin, oesophagus, stomach, ileum, colon, and rectum. The results showed that viral DNA was detected in the skin. The LC16m8 viral DNA was also detected in the ileum and rectum (Fig. 6c). No significant changes were observed in body weight, temperature, leucocyte count, or RBC levels; however, a slight increase in the white blood cells was observed (Fig. 6d and Supplemental Figure S5).

As an acute response, plasma CRP and CPK levels increased one-day post–challenge (Fig. 6e). Plasma cytokine levels indicative of acute inflammation responses, including IL-6, TNF-α, IL-12, IFN-γ, and chemokines, were measured (Fig. 6f and Supplemental Figure S6). IL-6 production peaked on day 3, while TNF-α, IL-12, IFN-γ, and chemokines levels peaked on day 7. The number of cytokine-producing myeloid cells was assessed. The percentage of the classical monocytes remained unchanged following the LC16m8 challenge; however, TNF-α and IL-6-producing classical monocytes were increased on day 7 and day 3, respectively (Fig. 6g and Supplemental Figure S7a). The percentage of nonclassical monocytes peaked on day 3 with IL-6-producing cells were increased from day 3 to 7. The induction of TNF-α-producing macrophages peaked on day 1, whereas IFN-γ-producing macrophages robustly increased on day 14 (Fig. 6g). The percentage of CD4^+^ T cells remained unchanged following the LC16m8 challenge. However, the percentage of CD8^+^ T cells peaked on day 7 (Supplemental Figure S7b). Cytokine production by CD4^+^ and CD8 T^+^ cells was also measured. The peak of IFN-γ-producing CD4^+^ and CD8^+^ T cells and TNF-α-producing cell, occurred on day 14, reflecting the induction of T-cell responses against LC16m8 (Fig. 6h).

## Discussion

The attenuated VACV strain LC16m8, initially approved in Japan for smallpox in 1975 and MPXV in 2022, was rigorously evaluated in preclinical models to assess its immunogenicity and safety. Since 2002, the Japanese government has stockpiled LC16m8 as a medical countermeasure against smallpox bioterrorism.^44^ Although routine smallpox vaccine has not been reinstated due to the absence of smallpox cases, the current PHEIC related to mpox may accelerate vaccination efforts, particularly among medical personnel and in endemic countries.

Our findings demonstrate that LC16m8 elicits robust immune responses in multiple preclinical models, including three mouse strains (BALB/c, C57BL/6J, and CAST/EiJ), non-human primates, and recently vaccinated humans. The vaccine induced strong antibody responses against VACV proteins, except for B5, which is not expressed in the vaccine. The response to MPXV was more selective, with notable induction of antibodies against H3, A35, and M1. This differential response highlights the vaccine's ability to target key antigens while avoiding those not present in the attenuated strain, offering insights for developing diagnostic tools for serological tests in populations vaccinated against or infected with VACV or MPXV.

In addition to humoural immunity, LC16m8 vaccination resulted in the induction of GC B cells and T_FH_ cells in the inguinal lymph nodes, critical for long-term immunity.^45^ Induction of these responses strengthens the immunogenic profile of LC16m8, suggesting that cellular immunity plays an essential role in protection against MPXV infection. Further, BALB/c exhibited stronger antibody and T-cell responses than C57BL/6J mice, indicating that strain-specific differences in vaccine immunogenicity should be considered when designing and deploying vaccines. Despite the robust GC B and T_FH_ cell induction in mice, the variability in the antibody responses across different viral proteins remains unexplained. Given that the MPXV genome encodes approximately 180 proteins, with limited data on the expression levels and immunogenicity, further research is required to map immunodominant epitopes and determine their correlation with protection against MPXV infection. Future studies incorporating TCR/BCR analysis and antigen profiling will be crucial to addressing this gap and refining our understanding of LC16m8-induced immunity.

Several studies have demonstrated the efficacy of LC16m8 against MPXV in non-human primate models. This study assessed its efficacy in CAST/EiJ mice, which are highly susceptible to MPXV infection. Following a single LC16m8 immunisation, antibody responses against both VACV and MPXV proteins were observed. Further, while body weight loss was comparable between vehicle-treated and vaccinated mice, LC16m8 immunised mice exhibited significantly lower viral loads in the lungs and reduced lung wet weights, indicating pulmonary infection and oedema. One mouse exhibited weaker antibody responses against VACV L1 and D8, and MPXV M1, correlating with detectable viral loads in the lungs, albeit at low levels. Given that M1 is the MPXV orthologue of VACV L1, our findings reinforce M1 as a promising vaccine antigen, in agreement with previous reports.^27^^,^^46^^,^^47^

The efficacy of LC16m8 translation from animal models to humans is critical for rapid vaccine development and has been investigated in a cohort of recently vaccinated individuals. Consistent with the murine data, the vaccine did not elicit antibody responses against B6; the orthologue of VACV B5 deleted in LC16m8. However, there was a marked increase in antibody responses against MPXV A35 in most subjects, along with specific T-cell activation, as demonstrated by the induction of CD4^+^ and CD8^+^ T cells. Importantly, LC16m8 vaccination enhanced the neutralising antibody activity against various MPXV strains, including those from different clades (Ia, IIa, and IIb). In addition, we synthesised A35 protein from the clade Ib strain. Although only a single mutation was found in clade Ib compared to Ia, the antibody responses were comparable. These findings suggest that LC16m8 could provide broad protection across multiple MPXV clades, which is a crucial feature given the genetic diversity and geographic spread of MPXV.

The safety of LC16m8 was further assessed in a non-human primates, where cynomolgus monkeys were intravenously challenged with a high dose of the vaccine strain. The results were encouraging, with pox lesions appearing on the skin by day 7, scabbing by day 14, and no mortality. Viral DNA was detected in the skin, ileum, and rectum; however, no significant changes in body weight, temperature, or haematological parameters were observed. Acute inflammatory responses, as indicated by increased levels of CRP, CPK, IL-6, TNF-α, IL-12, and IFN-γ, were observed, peaking at different time points, indicating a transient immune response. In addition, cytokine-producing myeloid cells and CD8^+^ T-cell activation were also detected, suggesting the vaccine's role in stimulating cellular immunity.

Preclinical and early clinical evaluations of LC16m8 highlight its potential as an effective tool for combating MPXV, particularly for outbreak containment. Several studies have demonstrated the efficacy of LC16m8 against the lethal MPXV Zaire strains.^42^^,^^43^ While mRNA vaccines have shown promise in non-human primates, comparisons indicate that both mRNA and attenuated vaccines provide comparable survival rates post–challenge.^47^ A major advantage of attenuated vaccines, such as LC16m8, is their ability to confer long-term protection following a single immunisation.^48^ The ability of the vaccine to elicit robust humoural and cellular immune responses, coupled with a favourable safety profile, makes it a viable candidate for broader deployment. However, further studies are needed to assess its safety in vulnerable populations, such as pox vaccine-naive children and immunocompromised populations, and to optimise formulations for enhanced immunogenicity across diverse demographic groups. The WHO PHEIC declaration for MPXV clade Ib further underscores the urgency of these efforts, calling for a coordinated global response to control and eventually eradicate MPXV.

The findings from the evaluation of LC16m8 provide important insights for the future design and development of MPXV vaccines. Given the genetic diversity and rapid evolution of MPXV, particularly with the emergence of distinct clades such as clade Ib, vaccine candidates must be designed to provide broad-spectrum protection. This may require the incorporation of multiple antigens or developing polyvalent vaccines targeting conserved regions across different MPXV strains. Furthermore, cellular immunity, particularly the induction of strong Th1-type responses and memory T cells, is crucial for long-term protection and should be prioritised in vaccine development.

Moreover, the safety profile of future vaccines must be carefully optimised to minimise adverse effects, especially in vulnerable populations, such as immunocompromised individuals. While attenuated vaccines like LC16m8 have shown promise, further research into alternative vaccine platforms, such as mRNA or protein subunit vaccines, could offer safer and more scalable options.^27^^,^^49^ Finally, global collaboration in vaccine development, testing, and distribution is essential to ensure that new vaccines are effective and accessible to high-risk populations, particularly in endemic regions of Africa.

In conclusion, the development and deployment of LC16m8 represent a major advancement in the global response to MPXV, with implications that extend beyond the immediate threat of mpox to broader strategies for managing emerging infectious diseases.

### Limitations

This study has several limitations. This study used samples from six volunteers who received the LC16m8 vaccine, limiting the statistical power and generalisability of the results. Routine smallpox vaccination was discontinued in 1976, making it challenging to obtain a larger cohort. Although 50 participants received LC16m8 in a recent clinical trial in Japan,^21^ we have obtained valuable new specimens and evaluate both the humoural and cellular immune responses. For humoural immune responses, we measured the antibody responses and neutralised antibodies against both VACV and MPXV. However, we were unable to assess neutralising responses against the clade Ib strain of MPXV due to the lack of available strains.

Regarding the cellular immune responses, we synthesised peptide pools of several VACV-derived proteins to evaluate T-cell activation. However, we did not observe CD4^+^ or CD8^+^ T-cell responses, despite previous studies demonstrating T-cell activities to these peptide pools.^50^

Another limitation of our study was the small number of monkeys used for the analysis (n = 4), which may have reduced the statistical power of our findings and limited the generalisability of the results. Although our analysis did not reveal significant differences between the endpoints (Supplemental Figure S10), the small sample size may have limited our ability to detect subtle effects, particularly when comparing immune responses between endpoints. Future studies with larger sample sizes are necessary to validate these findings and provide a more robust understanding of vaccine responses in non-human primates.

Future studies addressing these limitations through larger clinical studies, expanded antigen profiling, and alternative vaccine platforms will be crucial for optimising MPXV vaccine efficacy, safety, and global accessibility.

## Contributors

K.K., D.U., Y.K., E.S., F.Y., T. Okamura, T. Onodera, A.J.T., A.S., A.F.A., T.S., and J.T. performed the experiments and analysed the data. K.K., Y.K., E.S., F.Y., T. Okamura, T.S., and J.T. wrote the methods. K. K., K. S., Y. T., Y. Y., and K. J. I. conceived the project and designed the experiments. N.T.S., S.M., M.K., K.S., Y.T., Y.Y. and K.J.I. supervised this study. T.H., B.T., K.L., and H.N. provided intellectual advice and comments on the study. K.K. and K.J.I. wrote the manuscript. K.K. and K.J.I. accessed and validated the underlying data reported in this manuscript. All authors read and approved the manuscript.

## Data sharing statement

Any additional data reported in this paper is available from the corresponding author upon request.

## Declaration of interests

K.S. received consulting fees from Moderna Japan Co., Ltd. and Takeda Pharmaceutical Co., Ltd. and honoraria for lectures from Moderna Japan Co., Ltd. and Shionogi & Co., Ltd. The authors declare no conflicts of interest.

## Appendix A Supplementary data

Supplementary Table S1 and Figures S1–S10 Supplemental Figure 1. **Immunogenicity of LC16m8 strain between different administration routes**. (a) BALB/c and (b) C57BL/6J mice (n = 5 each) were subcutaneously (s.c.) or intraperitoneally (i.p.) immunised with the LC16m8 strain (2 × 10^6^ pfu). IgG1 and IgG2a/c antibody titres against A33, D8, and L1 were measured using ELISA 21 days post-immunisation. (a and b) Data are shown as the mean values. Statistical significance was assessed using the Kruskal–Wallis test, followed by Dunn’s multiple comparisons. Supplemental Figure 2. **Gating strategy of GC B cells and TFH cells in inguinal lymph nodes**. Lymph nodes were harvested from the vaccinated mice, and single-cell suspensions were prepared. The cells were gated for lymphocytes, singlets, live cells, and B or T cells. B cells were gated for GL7^+^CD38^+^ cells to identify GC B cells. T cells were further gated for CD4^+^CD8α^-^ to identify CD4^+^ T cells and for PD-1^+^CD185^+^ cells to identify T_FH_ cells. Supplemental Figure 3. **Gating strategy of activated-induced marker assay in human PBMCs**. After 16 h of incubation, human PBMCs were gated for lymphocytes, singlets, live T cells, and CD4^+^ or CD8^+^ T cells. CD4^+^ T cells were further gated for CD137^+^CD134 (OX40)^+^ cells to identify activated CD4^+^ T cells. CD8^+^ T cells were further gated for CD137^+^CD69^+^ cells to identify activated CD8^+^ T cells. Supplemental Figure 4. **Antibody responses by LC16m8 vaccination in human**. (a) Six healthy volunteers who received smallpox vaccine LC16m8. Blood samples were collected before and one month after vaccination and separated into plasma and PBMCs. (b) Plasma total IgG antibody against A27 (VACV) was measured using ELISA. Statistical significance was assessed using the binomial test in RStudio. Supplemental Figure 5. **Haematological analysis of LC16m8 strain challenged monkeys**. Red blood cell (RBC) and white blood cell (WBC) concentrations in the blood were measured using an automatic blood cell analyser (Sysmex). dpc: days post-challenge. Supplemental Figure 6. **Immune cell profiling of monkey PBMCs after LC16m8 strain challenge**. (a) The percentages of myeloid cells and cytokine-producing myeloid cells were analysed by flow cytometry. (b) Percentages of T cells and cytokine-producing CD4^+^ and CD8^+^ T cells were analysed using flow cytometry. dpc: days post-challenge. Supplemental Figure 7. **Plasma cytokine profile in cynomolgus monkey**. Plasma cytokine levels were measured using Luminex. dpc: days post-challenge. Supplemental Figure 8. **Gating strategy for monkey myeloid cells**. The PBMCs were gated to singlets, lymphocytes, singlets, CD45^+^, lineage^-^live cells, non-DCs, and macrophages. Classical and nonclassical monocytes were gated from the non-macrophage population. Nonmacrophages were gated to identify classical and nonclassical monocytes. Macrophages, classical monocytes, and nonclassical monocytes were further gated to identify cytokine-producing cells. Supplemental Figure 9. **Gating strategy for monkey T cells**. The PBMCs were gated for singlets, lymphocytes, singlets, CD45^+^, lineage^-^live cells, T cells, CD4^+^ or CD8^+^ T cells, and macrophages. CD4^+^ and CD8^+^ T cells were further gated to identify the cytokine-producing cells. Supplemental Figure 10. **The area under the curve analysis of the cynomolgus monkey dataset**. The area under the curve (AUC) was calculated for each monkey using data from days 0 to 10, which were the common time points across all monkeys. Following this calculation, the Mann–Whitney U test was performed to assess the statistical differences between each endpoint. This Figure presents the AUC values for each monkey and compares the datasets based on each endpoint difference.

Graphical Abstract
