# Supplementary figures and images for: Immunological analysis of LC16m8 vaccine: preclinical and early clinical insights into mpox
Source: eBioMedicine. 2025 Apr 15;115:105703. doi: 10.1016/j.ebiom.2025.105703 (PMC12020844; doi:10.1016/j.ebiom.2025.105703)

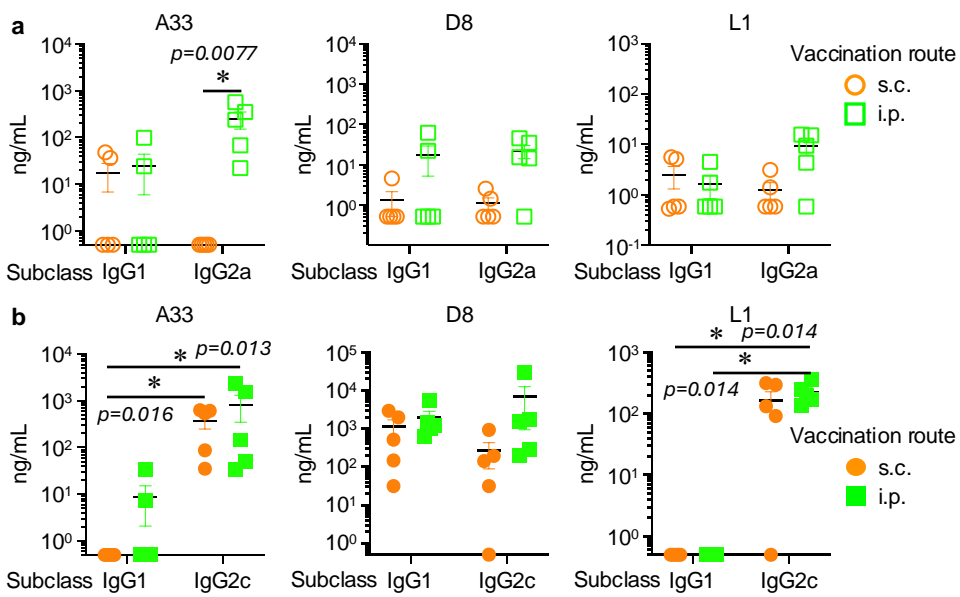

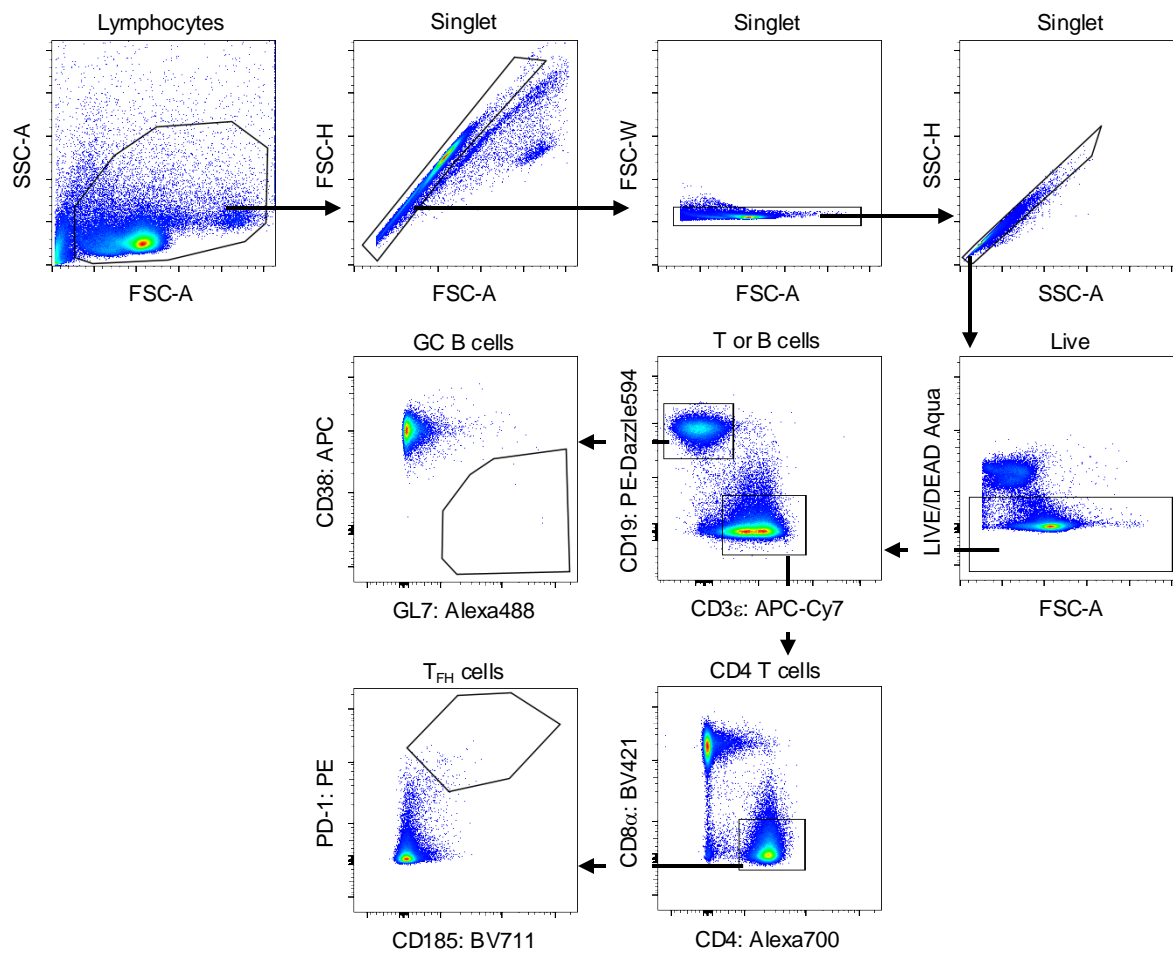

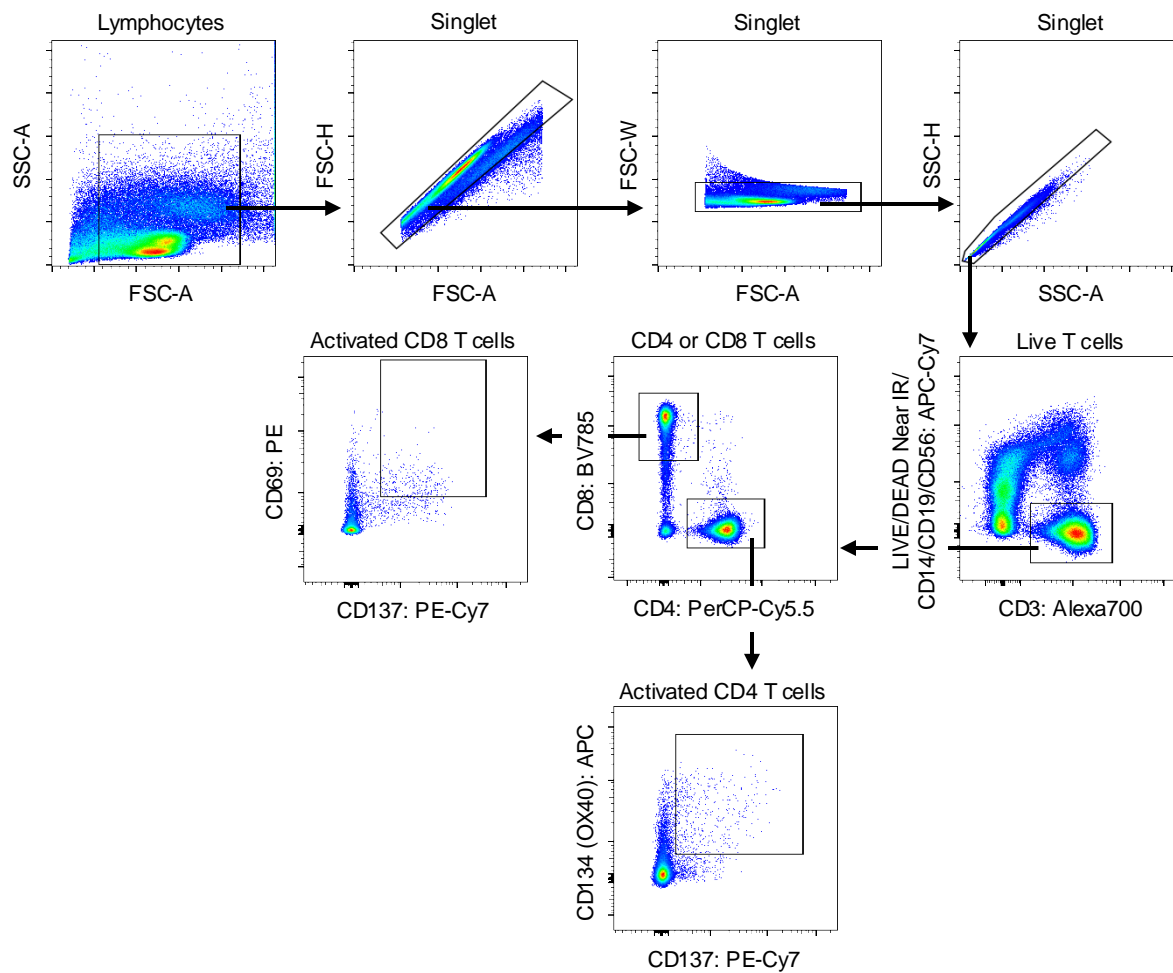

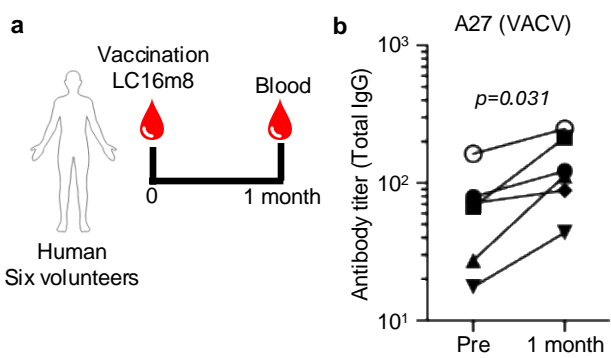

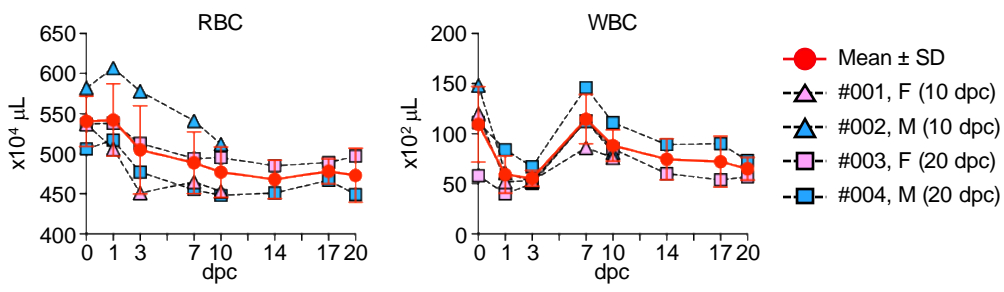

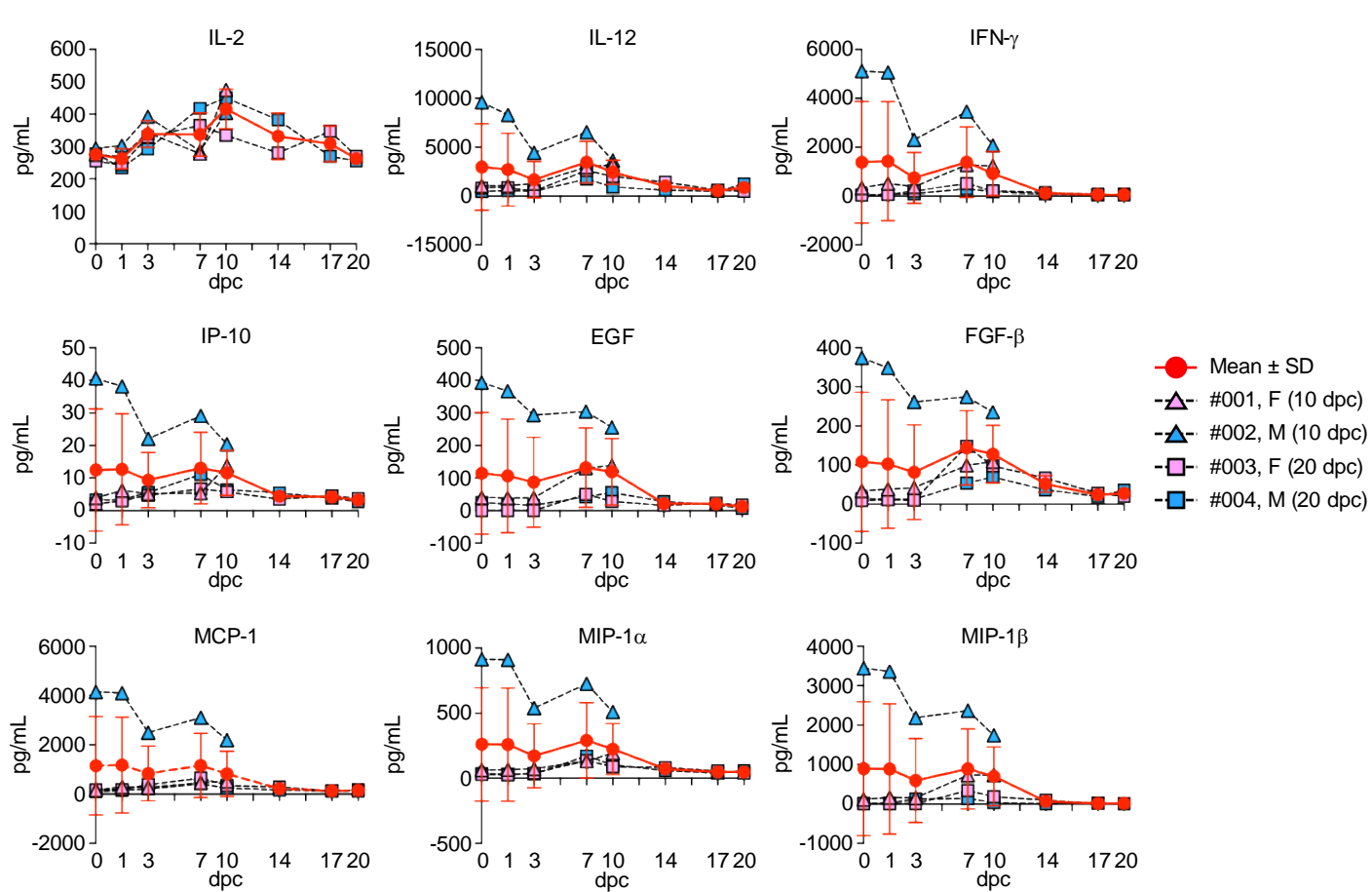



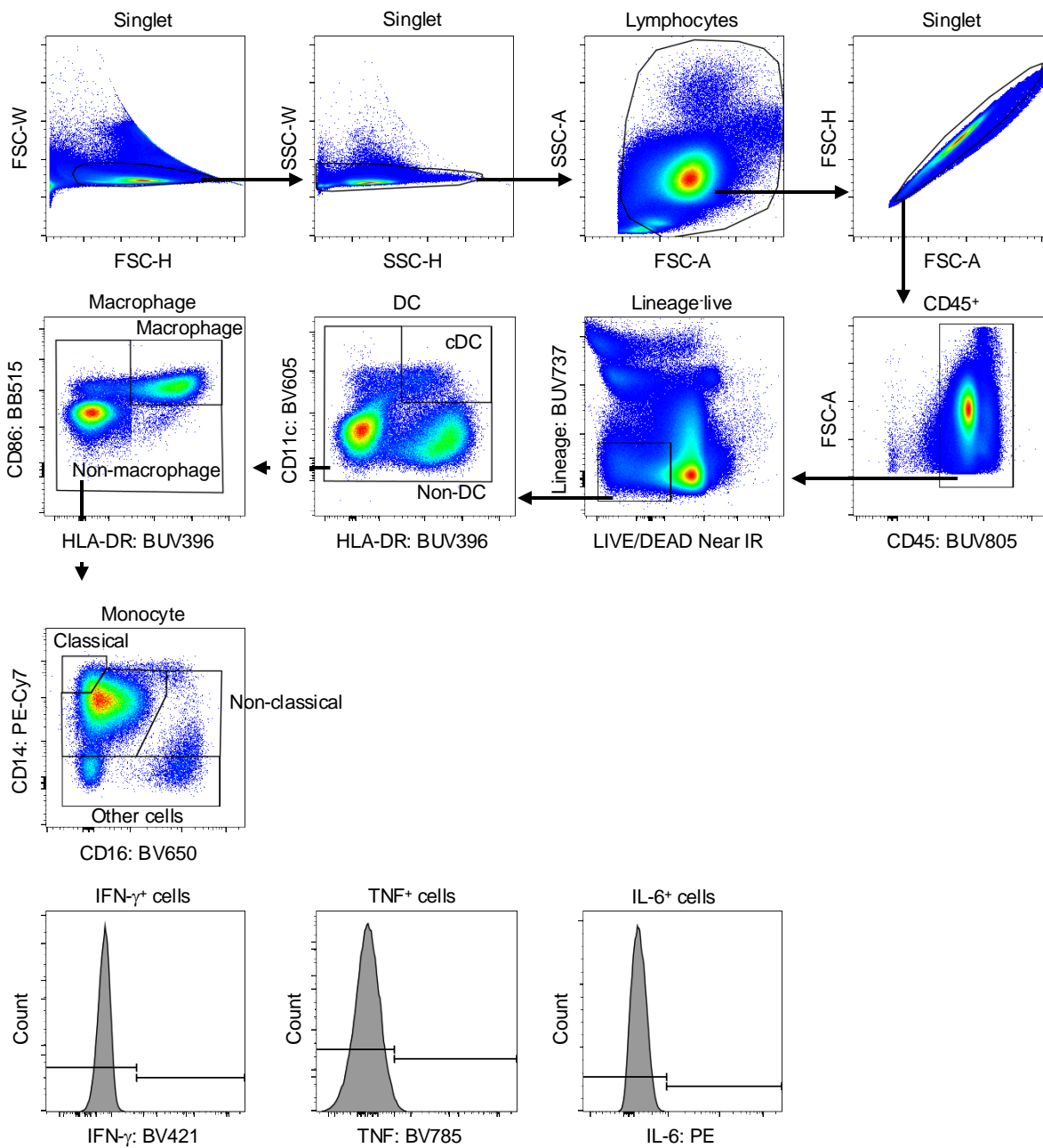

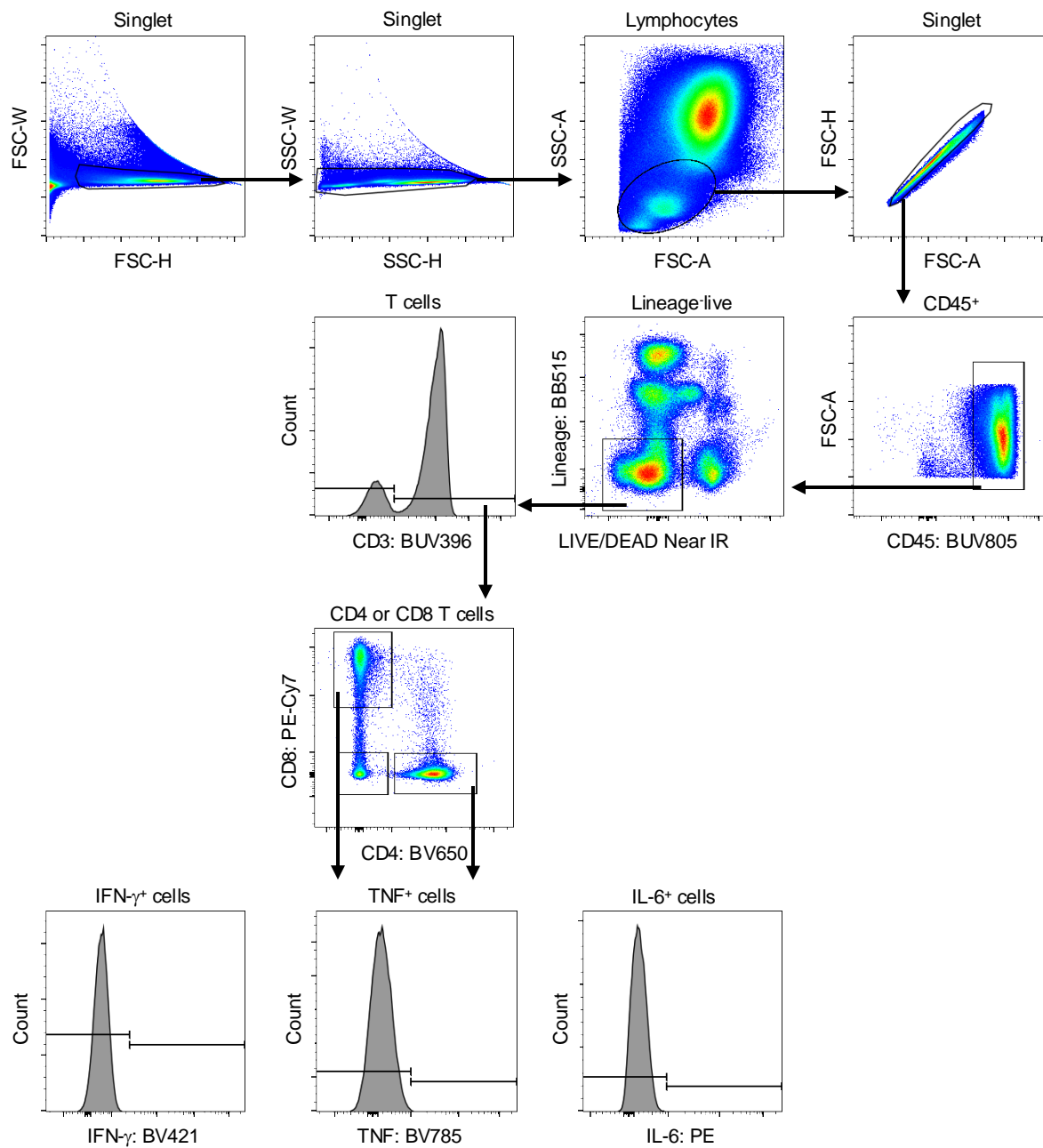

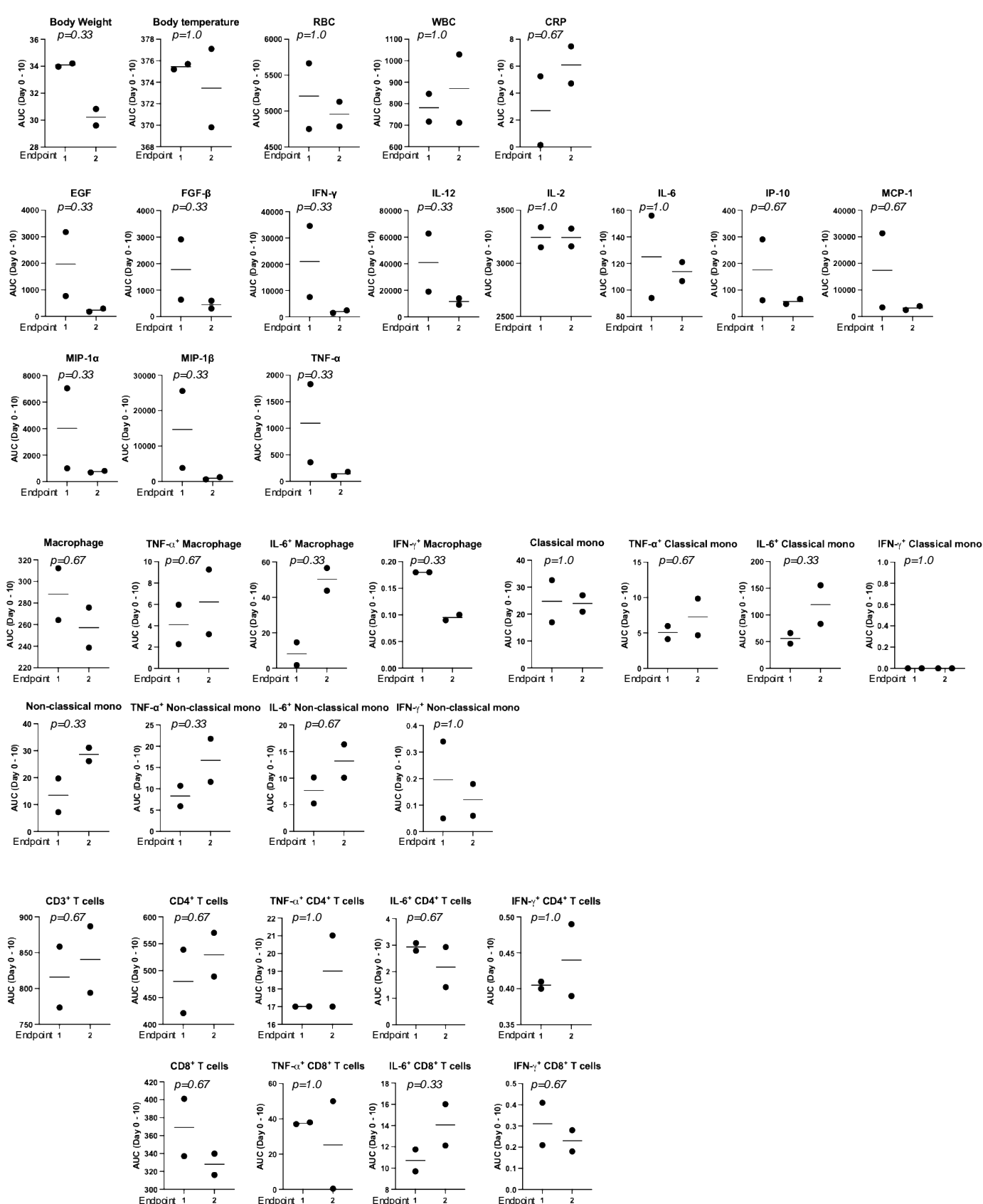

Supplement: Supplementary Table S1 and Figures S1–S10 — Supplemental Figure 1. Immunogenicity of LC16m8 strain between different administration routes. (a) BALB/c and (b) C57BL/6J mice (n = 5 each) were subcutaneously (s.c.) or intraperitoneally (i.p.) immunised with the LC16m8 strain (2 × 10^6^ pfu). IgG1 and IgG2a/c antibody titres against A33, D8, and L1 were measured using ELISA 21 days post-immunisation. (a and b) Data are shown as the mean values. Statistical significance was assessed using the Kruskal–Wallis test, followed by Dunn’s multiple comparisons. Supplemental Figure 2. Gating strategy of GC B cells and TFH cells in inguinal lymph nodes. Lymph nodes were harvested from the vaccinated mice, and single-cell suspensions were prepared. The cells were gated for lymphocytes, singlets, live cells, and B or T cells. B cells were gated for GL7+CD38+ cells to identify GC B cells. T cells were further gated for CD4+CD8α- to identify CD4+ T cells and for PD-1+CD185+ cells to identify TFH cells. Supplemental Figure 3. Gating strategy of activated-induced marker assay in human PBMCs. After 16 h of incubation, human PBMCs were gated for lymphocytes, singlets, live T cells, and CD4+ or CD8+ T cells. CD4+ T cells were further gated for CD137+CD134 (OX40)+ cells to identify activated CD4+ T cells. CD8+ T cells were further gated for CD137+CD69+ cells to identify activated CD8+ T cells. Supplemental Figure 4. Antibody responses by LC16m8 vaccination in human. (a) Six healthy volunteers who received smallpox vaccine LC16m8. Blood samples were collected before and one month after vaccination and separated into plasma and PBMCs. (b) Plasma total IgG antibody against A27 (VACV) was measured using ELISA. Statistical significance was assessed using the binomial test in RStudio. Supplemental Figure 5. Haematological analysis of LC16m8 strain challenged monkeys. Red blood cell (RBC) and white blood cell (WBC) concentrations in the blood were measured using an automatic blood cell analyser (Sysmex). dpc: days post-challenge. Supplementa [file mmc1.pdf]

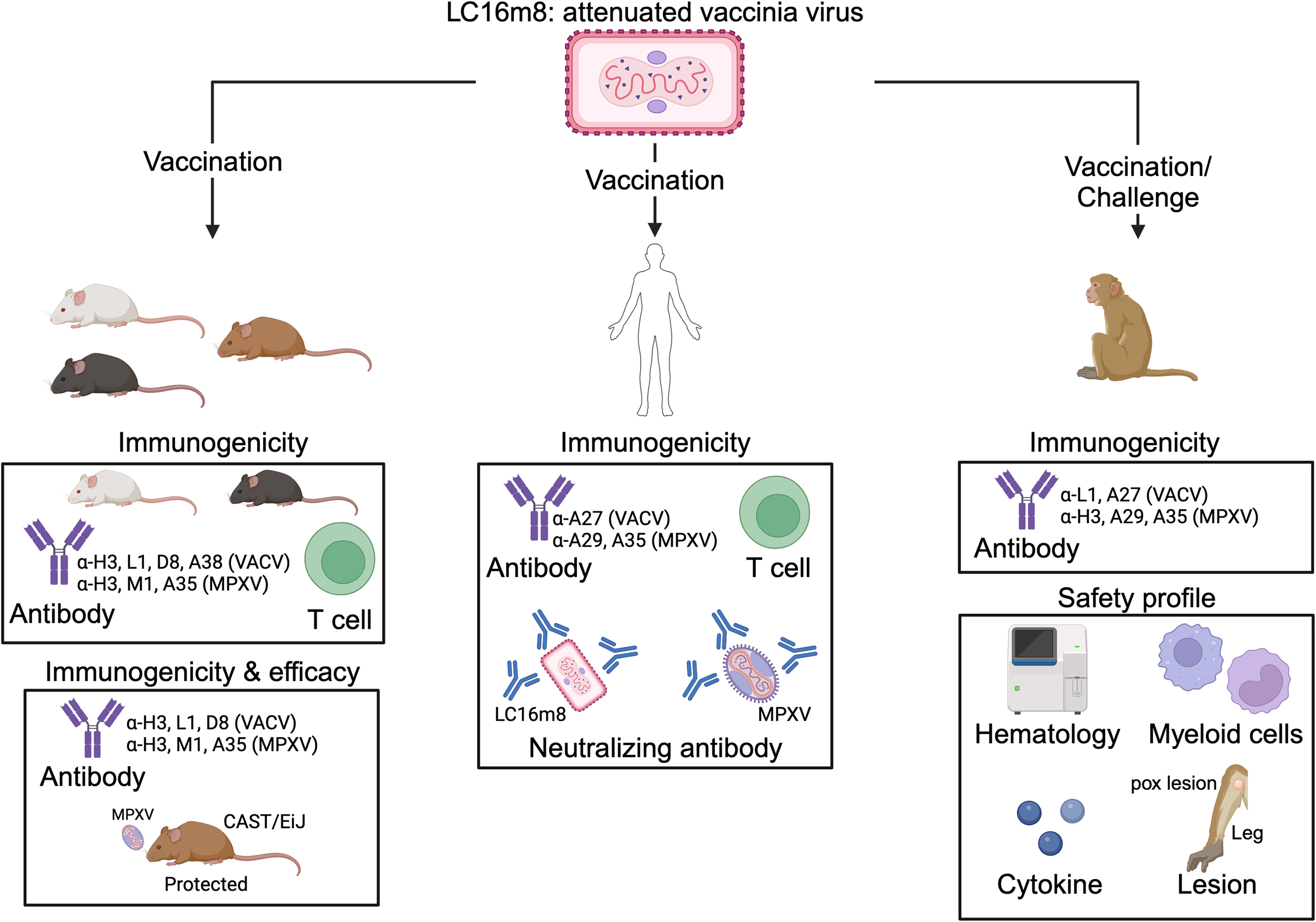

Supplement: Graphical Abstract [file figs1.jpg]
